# The utility of 3D models to study cholesterol in cancer: Insights and future perspectives

**DOI:** 10.3389/fonc.2023.1156246

**Published:** 2023-04-03

**Authors:** Thea-Leonie du Plessis, Naaziyah Abdulla, Mandeep Kaur

**Affiliations:** School of Molecular and Cell Biology, University of the Witwatersrand, Johannesburg, South Africa

**Keywords:** cholesterol, 3D culture, organoids, spheroids, cancer

## Abstract

Cholesterol remains a vital molecule required for life; however, increasing evidence exists implicating cholesterol in cancer development and progression. Numerous studies investigating the relationship between cholesterol and cancer in 2-dimensional (2D) culture settings exist, however these models display inherent limitations highlighting the incipient need to develop better models to study disease pathogenesis. Due to the multifaceted role cholesterol plays in the cell, researchers have begun utilizing 3-dimensional (3D) culture systems, namely, spheroids and organoids to recapitulate cellular architecture and function. This review aims to describe current studies exploring the relationship between cancer and cholesterol in a variety of cancer types using 3D culture systems. We briefly discuss cholesterol dyshomeostasis in cancer and introduce 3D *in-vitro* culture systems. Following this, we discuss studies performed in cancerous spheroid and organoid models that focused on cholesterol, highlighting the dynamic role cholesterol plays in various cancer types. Finally, we attempt to provide potential gaps in research that should be explored in this rapidly evolving field of study.

## 1 Introduction

Despite significant efforts and progress made by researchers globally, cancer is still one of the leading causes of mortality worldwide (1). The 2020 Global Cancer Observatory (GLOBOCAN) cancer statistics documented 19.3 million new cancer cases and approximately 10 million cancer-related deaths (2). The global cancer incidence is expected to increase by 47% from 2020 to 2040, with majority of cancer cases being observed in transitioning countries due to demographic changes and socioeconomic development (1, 2).

**Figure 1:**
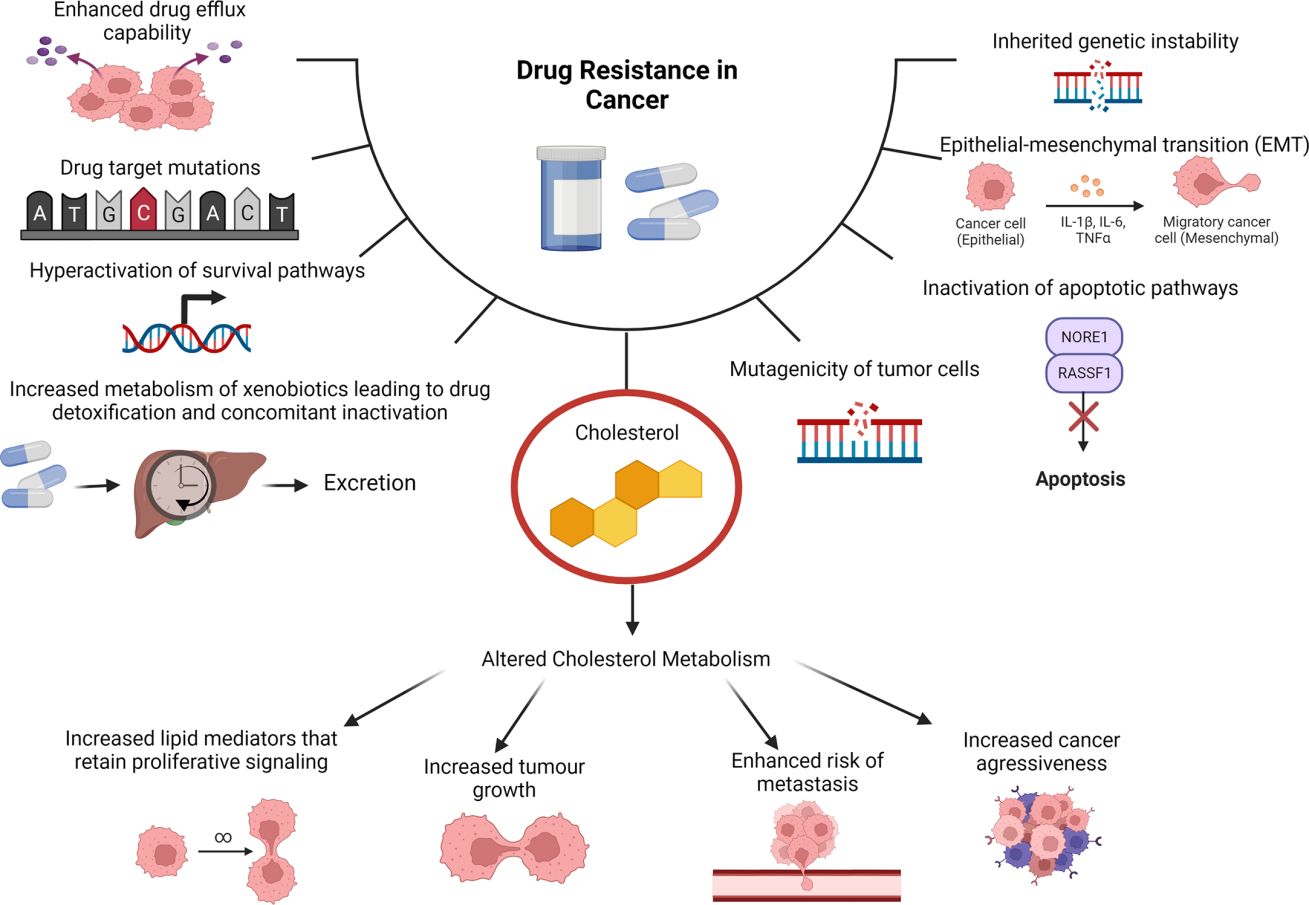
Cholesterol as a Contributing Factor to Drug Resistance. Molecular mechanisms of drug resistance in cancer include tumors having enhanced drug efflux capabilities, drug target mutations, hyperactivation of survival pathways, increased metabolism of xenobiotics leading to drug detoxification and concomitant inactivation can cause drug resistance in cancer. Additionally inherited genetic instability, epithelial-mesenchymal transition (EMT), inactivation of apoptotic pathways and mutagenicity of tumor cells. Cholesterol is also a contributing factor due to altered cholesterol metabolism that can cause increased lipid mediators that retain proliferative signaling, increased tumor growth, enhanced risk of metastasis and increased cancer aggressiveness.

Cancer is described as a heterogeneous disease as cancerous cells can acquire a broad range of capabilities during the multistep development into a tumor. These acquired capabilities are collated into 14 biological hallmarks, namely, sustained proliferative signaling, promoting inflammation, evading growth suppressors, angiogenesis induction, enabling replicative immortality, deregulated cellular energetics, activating invasion and metastasis (3). The additional hallmarks include avoiding destruction by the immune system, evasion of cell death, senescent cells, polymorphic microbiomes, non-mutational epigenetic reprogramming, unlocking phenotypic plasticity and lastly the ability to cause or allow genetic mutations that will contribute to genomic instability (4). Reprogramming of cholesterol metabolism is crucial to facilitating these hallmarks leading to increased cell proliferation, migration and invasion as well as drug resistance (5). Drug resistance is largely associated with the heterogeneity of cancer and its advanced capabilities to reprogram various signaling pathways in a highly efficient manner (6). The factors that mediate such resistance include suppression of apoptosis, altered drug metabolism, alteration of epigenetic and drug targets, enhanced DNA replication and repair systems and differences observed in individuals genetic makeup (**Figure 1**) (7). These factors work synergistically resulting in decreased efficacy of conventional therapeutics, which ultimately manifests in disease relapse.

Interestingly, as cancer develops, the cancerous cells particularly need to adapt their metabolic requirements to facilitate sustained growth. By altering various metabolic pathways, cancerous cells are able to successfully enable tumor initiation, promotion and progression, however, this is dependent on nutrient availability and tumor site (8). It has been established that lipid metabolism is often dramatically altered in cells undergoing neoplastic transformation as this allows for increased *de novo* lipogenesis, fatty acid uptake and oxidation, resulting in increased energy production and lipid accumulation (9). By altering lipid metabolism, cancerous cells can provide sufficient phospholipids for cellular membranes and lipid mediators that sustain proliferation and various signaling pathways respectively. A particular molecule of interest is cholesterol due to its various roles in the membrane and signal transduction pathways. As cancerous cells are highly proliferative, it can be suggested that an abundance of cholesterol can be used to fulfil the increased demand of substrates required for membrane biosynthesis and growth (10). Various enzymes and genes required for *de novo* cholesterol biosynthesis are upregulated in several cancer types indicating a link between cancer growth and development (9). Cancerous cells are also able to increase cholesterol storage and uptake ultimately compromising cholesterol homeostasis, specifically during cancer development and progression (10, 11).

Molecular mechanisms pertaining to cancer and metabolic processes such as cholesterol metabolism have largely been elucidated through 2D culture systems. However, these models provide an over simplified understanding of the complex pathways that occur within the body (12, 13). Furthermore, these model systems are cultured on hard plastics and cannot recapitulate chemical and mechanical cues that allow for cell-cell and cell-extracellular matrix (ECM) interactions (13). Often these models do not capture all the existing tumor subtypes due to high proliferation rates, variation in chromosome arrangements and alterations in gene expression upon high passage rates (14). As a result, drugs that are discovered are often those that target anti-proliferation instead of broad-spectrum anticancer compounds (14). Therefore, lately the focus has shifted to 3D culture systems such as spheroids and organoids. Spheroids are more complex than standard 2D cell culture as they are able to mimic tumor features and present with gene expression patterns closer to original tumors (15). Spheroids are derived from cells and form by spontaneous aggregation, which is then followed by the binding of cell surface integrins to the ECM (16). In contrast, organoids are 3D, self-organizing, organotypic cultures that are derived from stem cells or adult stem cells (17, 18). Organoids are also able to mimic biochemical and physical cues of tissue development and homeostasis of the corresponding *in vivo* organ from which they were derived and provide a more holistic representation of the cell-type heterogeneity in various organs (17, 18).

This review aims to summarize cholesterol metabolism and its role in cancer development and the result of dysregulated cholesterol metabolism with a particular focus on the use of spheroid and organoid cultures as models.

## 2 Cholesterol metabolism and dyshomeostasis in cancer

**Figure 2:**
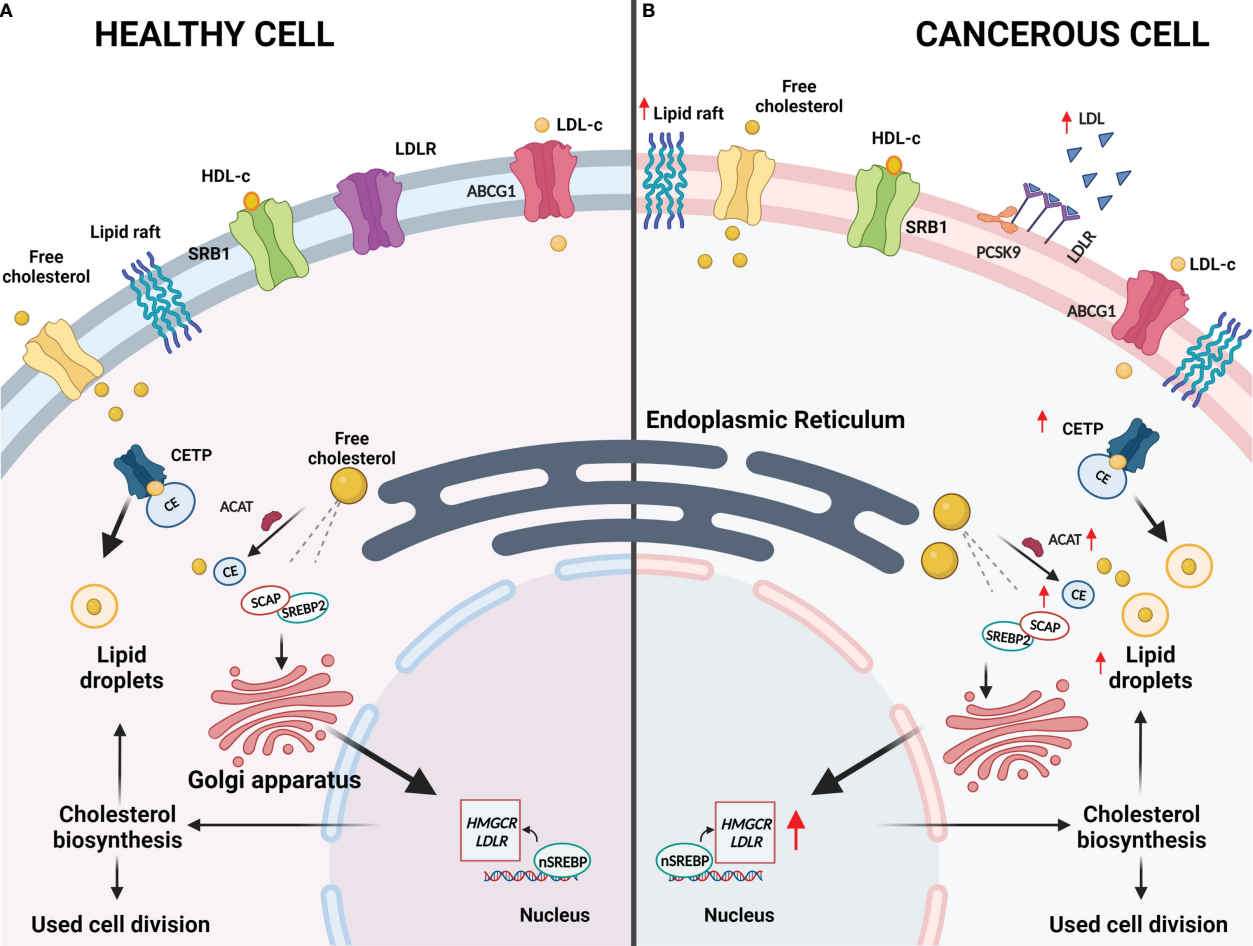
Deregulated Cholesterol Homeostasis in Cancer. **(A)** In healthy cells, normal cholesterol synthesis is required for various metabolic requirements. Cholesterol synthesis is initiated as citrate is converted to acetyl-coenzyme A and this is converted to lanosterol in a series of reactions that occur in the endoplasmic reticulum. Sterol regulatory element binding proteins (SREBPs) are a group of transcription factors that are responsible for regulating lipogenesis and lipid uptake. When cholesterol levels within the cells are low, cholesterol biosynthesis will be induced by SREBP2 and the lipid isoform (nSREBP2) will bind to the sterol response elements to trigger the expression of lipogenesis target genes, which include HMGCR, LDLR and PCSK-9. HMGCR catalyzes the rate-limiting step in cholesterol biosynthesis and LDLR imports cholesterol from the cellular environments, of which both will result in increased sterol levels within the cell. SREBP cleavage-activating protein (SCAP) is responsible for controlling intracellular biosynthesis of cholesterol, fatty acids and triglycerides whereas PCSK-9 is responsible for regulating LDLR by lysosomal degradation or inhibition of endocytic recycling of LDLR. To prevent excess accumulation of cholesterol within cells, acyl-CoA:cholesterol acyltransferase (ACAT) will convert excess free cholesterol into cholesterol esters (CEs). CEs will be shuttled by cholesteryl ester transfer protein (CETP) to lipid droplets that may be stored. Additionally, ATP-binding cassette subfamily C member 1 will transport cholesterol to HDL where cholesterol may be eliminated instead of being stored and scavenger receptor, class B type 1 (SRB1) will mediate the selective uptake of HDL-derived CEs into cells as required. **(B)** In cancerous cells, abnormal cholesterol synthesis is necessary to maintain their metabolic requirements. Cancerous cells can upregulate cholesterol synthesis to allow for rapid cell division and growth. HMGCR and LDLR are upregulated to ensure sufficient cholesterol biosynthesis and uptake, respectively, while ACAT and CETP will also be upregulated to ensure the excess cholesterol is safely stored. Lipid rafts have also been shown to be upregulated in cancerous cells to potentially allow for drug resistance, metastasis, and development due to the role that lipid rafts play in signaling.

Cholesterol is an important molecule in the human body and is biosynthesized by all mammalian cells (19, 20). This molecule is predominantly localized in cell membranes and is involved in the fluidity, rigidity and permeability of cell membranes as well as maintaining cellular homeostasis (19–21). Cholesterol is also able to bind to various transmembrane proteins, resulting in maintenance or alterations to their conformations, and it is able to interact with various sterol transport proteins that are capable of cholesterol transport and distribution (20, 22). Therefore, cholesterol is capable of modulating cell surface protein homeodynamics. Beyond the aforementioned roles, cholesterol is often associated with glycosylphosphatidylinositol (GPI) anchored proteins and sphingolipids, which form dynamic, microdomains termed lipid rafts that are crucial for cellular signaling (20, 22). A simplified representation of cholesterol synthesis and storage is illustrated in **Figure 2**. For a more comprehensive overview of cholesterol synthesis and homeostasis, the reader is referred to our previous publication (11).

Interestingly, cholesterol dyshomeostasis has become a key requirement for cancer initiation and progression whereby the synthesis and regulation of cholesterol is altered to meet the required metabolic demands of actively dividing cancerous cells (**Figure 2**) (23). Numerous studies are emerging, illustrating the roles of cholesterol dyshomeostasis in a variety of cancer types (24–27). One of the most widely studied regulators of cholesterol metabolism is sterol response element-binding proteins (SREBPs), which regulate the expression of 3-hydroxy-3-methylglutaryl coenzyme A reductase (HMGCR) (28, 29). SREBPs are able to promote HMGCR and low-density lipoprotein receptor (LDLR) transcription (29, 30). This facilitates increased cholesterol synthesis as well as increasing the intake of low-density lipoprotein (LDL), resulting in increased cholesterol within cells to facilitate increased cell proliferation and metabolism as required for tumor growth (29, 30). Furthermore, proprotein-convertase-subtilisin-kexin type-9 (PCSK9), which is responsible for regulating the LDLR, induces lysosomal degradation of LDLR resulting in increased LDL levels in the plasma membrane (31, 32). These increased levels of plasma LDL may result in hypercholesterolemia which could facilitate tumor cell growth (33).

Importantly, cells have developed various mechanisms to eliminate excess cholesterol as extremely high levels of free cholesterol in the cell membrane can change the physiochemical properties of the membrane, thereby affecting its function (34, 35). A relatively quick means of lowering the free cholesterol pool is through cholesterol esterification resulting in the formation of cholesteryl esters (CEs) (36). Cancer cells preferentially form CEs for storage and reuse in the form of lipid droplets, hence, it is hypothesized that this is a method in which cancerous cells are able to maintain and meet their required metabolic demands (35–37). Cholesterol esterification is facilitated by acyl-CoA:cholesterol acyltransferase (ACAT) and the cholesteryl ester transfer protein (CETP) is important for stabilizing CEs as well as promoting the storage of CEs as lipid droplets, which is less toxic to cells (38). Extracellularly, CETP mediates shuttling of CEs as well as triglycerides between high-density lipoprotein (HDL) and LDL. Alterations in the expression of ACAT and CETP have been documented in CE-rich breast cancer tumors (39). Furthermore, increased CE levels have been documented in pancreatic, breast and prostate cancer as well as glioma and leukemia (39). Additionally, CEs also potentiate phosphoinositide 3-kinase (PI3K)-dependent SREBP activity, which fuels the aggressiveness of cancer (36). These deregulations in cholesterol homeostasis facilitate the accumulation of cholesterol that result in poor patient prognosis and survival (21).

The dysregulation of cholesterol metabolism has significant implications on drug resistance. Cancerous cells are able to use cholesterol as well as oxygenated derivatives of cholesterol to facilitate drug resistance. This may be through signaling pathways that contribute to drug resistance or through directly affecting multi-drug transporters expression and activity (23). Additionally, cholesterol also forms part of various signal transduction pathways, particularly those involved in cell proliferation and survival, through lipid rafts which may facilitate cancer drug resistance, metastasis and cancer development (40, 41). This can be justified by the abundance of lipid rafts in cancer cells when compared to their healthy counterparts as well as the abundance of other proteins associated with malignant tumors (40, 41). There have been numerous studies that have illustrated that lipid rafts mediate cancer drug resistance (11, 41–45). These studies particularly identified signaling molecules associated with alterations in cholesterol, *via* depletion or synthesis, affecting migration, invasion, proliferation and apoptosis of cancerous cells (11, 41–45). For a detailed review on the association between cholesterol and drug resistance in cancer, the reader is directed to our previous publication (23). For a more extensive review on lipids and cholesterol with particular focus on cancer and therapeutic intervention, the reader is directed to the review by Kopecka and colleagues (46) and by Zalba and ten Hagen (47).

Various models have been employed to study the above-mentioned processes and have led to a greater understanding of cholesterol and its functionalities and pitfalls with majority of these studies being conducted on 2D *in vitro* cell lines. With the emergence of more robust 3D *in vitro* models, this potentiates further investigation into the dynamic role cholesterol has in a 3D setting.

## 3 3D models to study cancer

Spheroids are classified as spherical cancer cell aggregates that are self-assembled and can grow on low-attachment culture plates or in suspension culture (48). Growth of cancer cells as spheroids allows for them to be in close proximity to one another, forming a “mass”, allowing for the accumulation of cell-generated collagen that spheroids may use to anchor themselves (49). This allows spheroids to be integrated in a broad range of platforms, such as microfluidics and embedding in scaffolds to study a spectrum of diseases (15). Spheroid models are more complex than standard 2D cell culture due to their advanced capabilities that allow them to mimic tumor features, such as cell-cell and cell-ECM interactions, physio-chemical gradients have also been observed and gene expression patterns are much closer to original tumors than 2D cell lines (15). The ECM is also capable of forming cell-binding sites that are capable of controlling cell adhesion and migration (50). There are also differences in physical and physiological properties between 2D and 3D cultures (50, 51). This affects their sensitivity to drugs, with 2D cells being more sensitive in comparison to their 3D counterparts. This is due to 2D cells being unable to maintain normal morphology in comparison to 3D cell culture as well as there is differences in the organization of surface receptors present on the cells (50).

**Figure 3:**
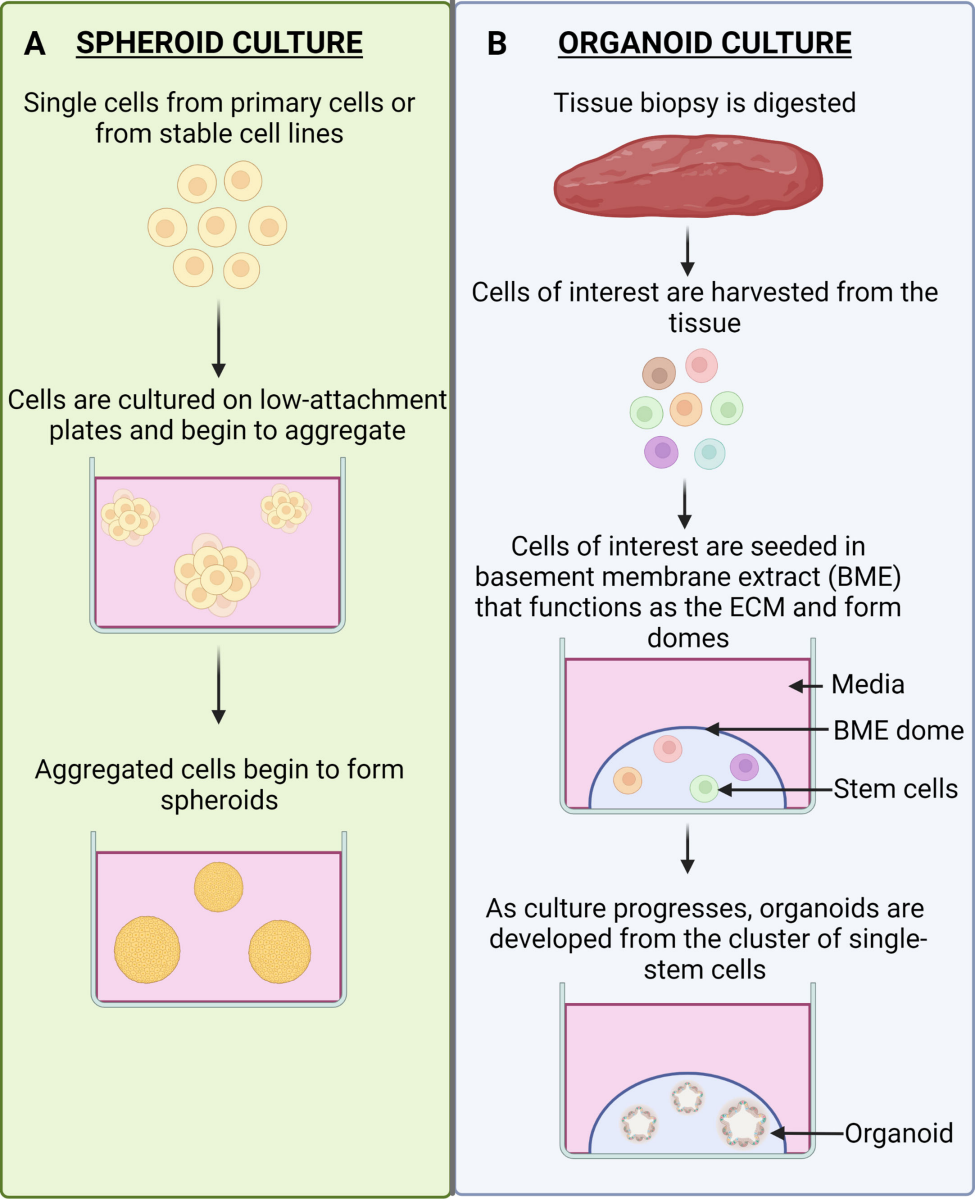
Spheroid Culture vs. Organoid Culture. **(A)** Spheroid culture. Spheroids are cultured by obtaining single cells from primary cells or from stable cell lines. The cells are naturally able to form aggregates and can do so in suspension culture. The aggregates will become more defined spheroids as the culture progresses. **(B)** Organoid culture. Tissue that is biopsied from patients will be digested into cells and the cells of interest can be harvested. These cells are then seeded in a ECM-mimicking substrate (BME), which will result in the formation of domes. The stem cells will be present in the domes and as the culture progresses it will give rise to organoids.

Organoids are well-known as 3D, self-organizing, organotypic cultures that have been derived from pluripotent stem cells or adult stem cells and are able to mimic its corresponding *in vivo* organ (17, 18). They are grown in culture in a basement membrane matrix and further supplemented with various growth factors to mimic the stem cell niches and provide a representation of the differentiated cell-type heterogeneity in particular organs (17, 18). The brief culture methods for spheroids and organoids are illustrated in **Figure 3**.

Organoids can also be derived from patients, making them patient specific, and can be obtained from any individual suffering from a variety of carcinomas (52). Organoid applications include, but are not limited to, drug screening, biobanking, to study rare cell lines, modelling disease development, pre-clinical models and to further personalized medicine (12, 18, 53–55). Organoids allow for cells to retain their natural shape and have been shown to resemble the organs from which they have been grown from (56). By using stem cells to recapitulate whole organs in the form of organoids, they aid in studying the mechanisms of drug metabolism, cell differentiation and expression levels that closely resemble the organ (56–58). Importantly, organoids can be used to overcome the challenges of gene and protein expression level variations, as they closely resemble those that would be observed *in vivo* (58). Furthermore, organoids as a 3D culture system has led to new insights in the field of drug discovery as drug molecules interact with the ECM and a large proportion of drug discovery relies on this interaction (58). Using organoids, that are grown in reconstituted basement membrane matrix, may allow researchers to better understand the metabolism and the effect drug molecules have on particular tissues and organs (58). By using organoids in drug discovery, cells that are present in these organoids retain their physiological properties and maintain a normal morphology. For a summary related to the advantages and limitations of the aforementioned models, the reader is directed to **Table 1** below.

**Table 1:** Comparisons summary of 2D culture and 3D culture.

	Models			
	2D Culture	3D Culture		References
	Cell Culture 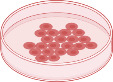	Spheroids 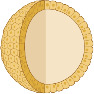	Organoids 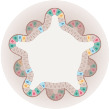	
*Accessibility*	More easily accessible as well-established culture reagents and cell lines easily available.	Accessible due to cellular sources and reagent availability.	More novel than the previous two methods, accessibility remains limited due to specific reagent suppliers and tissue availability from patients.	(59)
*Cost to maintain cultures*	Relatively cheap to maintain as commercially available reagents and assays.	Lower cost in comparison to organoids, but requires time to initiate and develop cultures.	More expensive, fewer commercially available tests and reagents. Can also be quite costly to initiate and develop cultures.	(58, 60)
*Well-established culture protocols*	Well-established and standardized protocols available for a variety of cell lines.	There are a number of well-established protocols (suspension culture, scaffold culture, etc) but use may be dependent on the type of experiments	Some well-established protocols available but no standardized protocols.	(56, 59, 61)
*Time expenditure to establish culture*	Within a few hours to a few days	Within a few days to a few weeks	Within a few weeks to a few months	(62–64)
*Patient-specific* & *cellular sources*	Yes, can derived from an established single cell-line or from patient primary cells	Yes, can be derived from patient primary cells or established cell-lines can be used	Yes, patient samples can be digested and specific cell-types harvested to generate organoids	(16)
*Cellular characteristics*	Cells often present with a flat and elongated shape. Cells are grown as monolayers and can only expand in two-dimensions	Cells are able to maintain their natural 3D shape and are able to form multiple layers and grow in three-dimensions as it would occur *in-vivo*		(60, 65–68)
*Cell proliferation rates*	Cell proliferation occurs at an unnatural rate and often cells are at the same stage of growth	Cell proliferation occurs at more natural rates and cells are sometimes at different stages of growth (as seen *in-vivo*)		(67, 69)
*Gene and protein expression levels*	Expression levels are vastly different when compared to *in-vivo* systems	Expression levels have been found to resemble those seen *in-vivo*		(60, 66, 67, 69)
*Sensitivity to drugs*	Cells present with minimal resistance to drugs, which can result in successful outcomes to administered drugs	Often present with more resistance to administered drug treatments, showing a more holistic overview to drug metabolism *in-vivo*.		(67, 70, 71)
*Tumor microenvironment*	Single cell types are often cultured without additional cell types, there is limited tumor heterogeneity, there is often no ECM substance and cell-cell interactions is limited to between one cell type. Therefore, does not accurately represent the tumor microenvironment.	Multiple cell types can be cultured together, there is better tumor heterogeneity observed, ECM substrate is present and cell-cell interactions can be observed between different cell types. Therefore, the tumor microenvironment is more accurately represented.		(72, 73)
*Usage, analysis* & *reproducibility*	Easily used to generate results and analysis is easily interpretable. Results are also easily reproducible	Usage can vary from user-to-user and from experiments, analysis can also be difficult to perform due to 3D nature and reproducibility remains an issue		(56, 74, 75)

## 4 The utility of 3D models in studying molecular mechanisms of cholesterol in cancer

As mentioned above, the complex role cholesterol plays in cancer development, progression, and drug resistance through its association with various signaling pathways has been documented in published literature. Granted the difference in properties of cells cultured in 2D vs 3D it is not surprising that 3D cultures would display altered metabolic processes and hence present with different physiological outcomes when compared with 2D cultures. Interestingly, emerging experimental evidence implicates cholesterol as a crucial metabolite in supporting the growth and malignancy of cancer cells in 3D. Seeing that 3D models present with a more accurate depiction of human pathophysiology these models are increasingly being employed. The use of spheroid and organoid models is currently employed in many translational applications to delineate the role of cholesterol in cancer. This will be expanded on below.

### 4.1 Cholesterol contributes to the proliferative and malignant potential of 3D cultures

Reprogramming of cellular energetics is emerging as a crucial hallmark that cancer cells subvert to facilitate disease progression. Upon carcinogenesis, the finely tuned mechanisms of cholesterol regulation are altered based on the energetic requirements of actively dividing cancerous cells. A fine example of this is documented in epithelial ovarian cancer (EOC), where these cancer cells are inherently sensitive to the levels of cholesterol with the existence of an upper threshold (76). When this threshold is surpassed, excessive levels of cholesterol can suppress tumour growth. This is evidenced by reduced spheroid growth and migratory capacity upon depletion of ATP binding cassette subfamily A member 1 (ABCA1) (76). Authors consequently postulate the potential of exploiting this cholesterol dependency for cancers that are susceptible to cholesterol-rich environments.

Interestingly, evidence exists indicating differential regulation of cholesterol metabolism in cells when cultured in 2D vs 3D. To illustrate this a recent study conducted by Byutaite and Petrikaite (77) demonstrated the differential response to statin treatment when breast cancer cells were cultured in 2D vs 3D spheroids. When both MCF-7 and MDA-MB-231 cell lines were cultured as monolayers, treatment with various statin classes had a significant effect as opposed to when these cell lines were cultured in 3D (77). In spheroids, the increased concentrations of lovastatin and simvastatin halted growth of MDA-MB-231 cells and increased concentrations of mevastatin and pitavastatin impeded growth of MCF-7 cells (77). Interestingly, in prostate cancer, gene expression profiles associated with epithelial to mesenchymal transition (EMT) differed following rosuvastatin treatment in 2D vs 3D cultures thereby potentiating greater insights from studying the cellular EMT process in a spheroid culture system as opposed to as an adherent monolayer (78).

Furthermore, the importance of cholesterol in colorectal cancer (CRC) tumorigenesis cannot be understated. Wang and colleagues, (79), demonstrated that cholesterol acts as a mitogen for intestinal stem cells (ISCs). Using mice models and intestinal organoids, authors elucidate that increased cellular cholesterol content *in vivo* (dietary cholesterol or cholesterol synthesis) stimulates crypt organoid formation by promoting ISC proliferation. Authors further implicated the phospholipid remodeling enzyme lysophosphatidylcholine acyltransferase 3 (Lpcat3) in increased cholesterol membrane saturation and activation of the cholesterol biosynthesis pathway thereby promoting proliferation of ISCs (79). It was further hypothesized that post-transcriptional activation of SREBP2 is likely the causative agent of enhanced expression of cholesterol biosynthesis (79). In another study, Wen and colleagues (80) documented that silencing of SREBPs 1 and 2 led to a significant alteration in metabolism affecting glycolysis, mitochondrial respiration as well as fatty acid oxidation (80). Furthermore, silencing of SREBPs led to a significant reduction in spheroid formation efficiency. This was justified by the reduced expression of cancer stem cell (CSC)-related genes including *CD44, CD133*, leucine-rich repeat containing G protein-coupled receptor 5 *(LGR5)*, and AXIS inhibition protein 2 (*AXIN2)* (80). This study hence implicated SREBP-dependent lipid biosynthesis in CRC tumorigenesis. Several studies support this view highlighting increased activity of the mevalonate pathway in CSCs of CRC. In the same light, it has been hypothesized that crosstalk exists between polyamine metabolism and the cholesterol synthesis pathways *via* SREBP2 which govern the proliferative and malignant potential of cells (81). In DLD-1 spheroids administering spermine (polyamine synthesis inhibitor) APCHA, inhibited 25-hydroxycholesterol (25-HC)-induced apoptosis in a SREBP2 manner. This inhibition resulted in increased transcriptional activity of SREBP2 and reduced apoptosis in DLD-1 spheroids (81).

In addition to biosynthesis, recent studies have implicated the cholesterol transporter ABCA1 in CRC disease aggressiveness. This is evidenced by the increased mitotic population and invasive potential in spheroids overexpressing ABCA1 (82). The malignant potential conferred on spheroids through ABCA1 overexpression is reliant on caveolin-1 stability which functions as a focal adhesion regulator (82). ABCA1 overexpression could facilitate increased levels of plasma membrane cholesterol which would consequently affect membrane composition, fluidity, and flexibility to confer spheroids with invasive potential (83). Furthermore, evidence exists implicating cholesterol efflux pump ATP-binding cassette subfamily G member 1 (ABCG1) in modulating tumor growth of rapidly metastatic colon cancer (84). Using tumor organoids, ABCG1 was shown to be elevated within the stemness-enhancing tumor environment, in contrast to ABCG1 depletion that lowered cellular aggregation and tumor organoid growth (84). Decreased tumor organoid growth was attributed to the autocrine accumulation of cytotoxic extracellular vesicles. Seeing that redundant and toxic substance accumulation can facilitate tumor regression, targeting ABCG1 could be viewed as a novel therapeutic strategy to eradicate CRC (84).

Pancreatic cancers display an inherent reliance on cholesterol uptake and metabolism to facilitate proliferation and viability. Several studies conducted emphasize the importance of cholesterol in pancreatic cancer (85–87). The mevalonate pathway has been implicated in niche-factor mediated proliferation and differentiation in pancreatic ductal adenocarcinoma (PDAC) organoids. Furthermore, niche factor dependency showed a correlation with tumor-differentiation grade (88). On this basis, authors proposed the use of subtype-based therapeutic strategies to combat PDAC (88). In a different study, Oni and colleagues (89), illustrated using pancreatic organoids, that sterol *O*-acyltransferase 1 (*SOAT1*) is fundamental to sustain the mevalonate pathway by converting cholesterol to chemically inactive CEs consequently promoting PDAC progression (89). PDAC has been described as an extremely lethal malignancy with a dismal prognosis and lack of effective therapies. Moreover, tumor suppressor protein 53 (p53) mutant PDAC cells that have undergone p53 loss of heterozygosity display mevalonate pathway dependency, where silencing of *SOAT1* downregulates the expression of mevalonate pathway genes and affects cell proliferation and tumor progression (89). This highlights a potential therapeutic target for PDAC by inhibiting *SOAT1*. In addition to *SOAT1*, the cholesterol uptake regulator Niemann-Pick C1-Like 1 (NPC1L1) is significantly upregulated in PDAC. Utilizing a classical patient-derived xenograft (PDX) model authors demonstrated an upregulation of genes involved in lipid metabolism and cholesterol homeostasis namely peroxisome proliferator-activated receptor gamma (*PPARG)* and nuclear receptor subfamily 1 group H member 3 (*NR1H3)* (LXRα) potentiating cross talk between stromal and cancer cells leading to cholesterol uptake (90). Consequently, treating PDAC organoids with ezetimibe (NPC1L1 inhibitor) significantly affected organoid growth and viability, making NPC1L1 a potential therapeutic target in PDAC (90).

Coupled with cholesterol, the metabolites of cholesterol also play a role in tumor progression. Cholesterol sulphate is one such metabolite synthesized by the enzyme sulfotransferase family 2B member 1 (SULT2B1b). In prostate cancer organoids, the expression of SULT2B1b inversely correlates with cancer progression (91). SULT2B1b is further associated with characteristics of malignant prostate cancer. Interestingly, a strong positive correlation between SULT2B1b and epithelial differentiation was also documented (91).

It can be noted that culturing of cells in 3D has facilitated novel insights into the role of cholesterol in cancer progression and malignancy. Several processes are subverted by cancer cells including those involved in cholesterol biosynthesis, influx, metabolism, and efflux. Importantly, the differential regulation of cholesterol metabolism in 2D vs 3D cultures highlights the incipient need to further study processes linked to cholesterol metabolism in 3D cultures as findings reported from 2D settings might not accurately mimic physiological conditions. Importantly, even though cancer cells are dependent on cholesterol, an inherent limit does exist where excessive levels of cholesterol can suppress tumor growth. In some cancers active mechanisms exist to convert excess cholesterol to chemically inactive CEs to promote tumor progression. Consequently, exploring further mechanisms of cholesterol regulation in 3D cultures is an area that should be actively pursued to delineate the role of cholesterol in cancer. Based on the multifaceted role of cholesterol in the cell, we attempt to provide a mechanistic view of cholesterol in cancer by elaborating on the transcriptomic alterations observed in 3D models.

### 4.2 Alterations to the cellular cholesterol transcriptome in 3D cancer models

Alterations to the transcriptome proves crucial in delineating the role of functional elements of the genome to disease pathogenesis (92). Importantly, understanding the underlying molecular dynamics that dictate cancer initiation and progression is pivotal to developing effective therapies for cancer eradication. Subsequently, culturing of 3D spheroid and organoid models have proved useful in elucidating alterations to the cholesterol transcriptome that consequently mediate cancer pathogenesis.

In a study employing tumor spheroids derived from breast cancer, culturing cells in a 3D environment, led to a significant alteration in the transcriptome of breast cancer cells with one of the major changes being deregulated cholesterol homeostasis (93). These findings are supported by a study conducted by Ehmsen et al. (94), where authors delineated the crucial role cholesterol plays in the propagation of triple negative breast cancer (TNBC) stem cells (94). TNBC cells cultured as mammospheres display increased expression of several genes involved in cholesterol biosynthesis including 3-hydroxy-3-methylglutaryl-CoA synthase 1 (*HMGCS1)*, farnesyl diphosphate synthase *(FDPS)*, farnesyl-diphosphate farnesyltransferase 1 *(FDFT1)*, lanosterol synthase *(LSS)*, NAD(P) Dependent Steroid Dehydrogenase-Like *(NSDHL)*, emopamil binding protein *(EBP)*, and 7-dehydrocholesterol reductase *(DHCR7)* (94). Importantly, a significant correlation was observed between high expression of these genes (5 out of 7 except, *LSS* and *DCHR7*) and shorter relapse-free survival in the basal-like breast cancer cohort. Moreover, treating these mammospheres with statins led to a decrease in mammosphere growth and formation efficiency hence potentiating targeting cholesterol biosynthesis as an attractive therapeutic for TNBC stem cells (94). Furthermore, Dattilo et al. (95) demonstrated that the cholesterol biosynthesis pathway was transcriptionally activated in TNBC mammosphere models and proved essential in facilitating cell survival and migration. Increased expression of squalene epoxidase (*SQLE)* and Mevalonate Diphosphate Decarboxylase (*MVD*) genes in the mammosphere model correlated to increased expression of these genes in a TNBC cohort, which was associated with lower probabilities of relapse-free survival and distal metastasis-free survival consequently linking this pathway to aggressive TNBC. Importantly, elevated expression of *SQLE* and *MVD* was documented as a common signature in all breast cancer subtypes, potentiating the significance of cholesterol as a therapeutic target in breast cancer (95).

Mechanistic insights into the role of cholesterol in CRC disease pathogenesis is provided by Seo et al. (96), documenting increased expression of genes and proteins *HMGCR, FDPS*, geranylgeranyl diphosphate synthase 1 *(GGPS1)*, and *SQLE* in tumor spheroids when compared to 2D adherent cultured cells (96). This was further validated by employing a clustered regularly interspaced short palindromic repeats (CRISPR) dropout screen with identification of *HMGCR, FDPS*, and *GGPS1* as top-ranked essential genes in 3D CSC-enriched spheroids (76). Corroborating findings from spheroid models, an upregulation of *SQLE* is also documented in CRC patient-derived organoids, which is associated with poor prognosis. Mechanistically, SQLE was shown to promote CRC through the accumulation of calcitriol and stimulation of CYP24A1-mediated mitogen-activated protein kinase (MAPK) signaling *in-vitro* and *in-vivo* (97). SQLE levels have been shown to correlate with tumor stage indicating a pathological association of SQLE with colorectal cancer initiation but not metastasis (97, 98). Furthermore, treatment with a SQLE inhibitor terbinafine markedly decreased organoid number and viability (97). As SQLE is the second rate-limiting enzyme in the cholesterol biosynthesis pathway, this highlights SQLE as a potential therapeutic target for early stage CRC (99).

Additionally, an upregulation of cholesterol biosynthesis genes was documented in organoids and primary colon tumors (100). HMGCR is crucial for the survival and pluripotency of the organoids as uncovered by CRISPR screens. Further studies implicated transforming growth factor beta (TGF-β) signaling as the mechanistic link between increased cholesterol biosynthesis and colon CSC-enriched spheroids survival. Furthermore, positive correlations between stemness markers (ephrin receptor B2 (EphB2) and CD44) and cholesterol-biosynthesis genes (*HMGCR, HMGCS1, FDPS, and FDFT1*) were documented (76). While Seo et al. implicate mevalonate-pathway derived prenylation moieties in stemness potential, Gao et al. in addition to prenylation, document a crucial role for cholesterol in stemness (76, 96) potentiating further investigation into the role of cholesterol in CRC.

While several of the aforementioned studies explored one tissue type, the development of organoid models has more recently proved useful in modelling tissue dependent cholesterol-related transcriptomic changes in response to a therapeutic intervention. To illustrate this, Rodrigues and colleagues (101) investigated the mechanistic actions of gefitinib (tyrosine kinase inhibitor), in human colon and small intestine organoids. It was observed that these presented with opposite gene expression signatures following treatment (101). The small intestinal organoids displayed activation of cholesterol biosynthesis (increased expression of *HMGCR, HMGCS1* and *CYP51A1*) in contrast to colon organoids where efflux (increased expression of *ABCA1*) was enhanced (101). The difference in metabolic response following treatment was attributed to the activation of AMP-activated protein kinase (AMPK) signaling in the colon but not the small intestine (101). Intriguingly, colon organoids were more sensitive to gefitinib in comparison to the small intestinal organoids, which suggests that increased cholesterol synthesis could serve as a potential mechanism to enhance resistance to gefitinib (101). Authors hence proposed that co-administration of chemotherapeutics and drugs targeting cholesterol may affect drug sensitivity and toxicity (101).

From a 3D culture perspective, an association between cholesterol and prostate cancer aetiology has also been documented. Co-culturing of prostate cancer spheroids with cancer-associated fibroblasts (CAFs) is seen as a reliable model for anti-androgen resistance. Neuwirt and colleagues (102), identify that CAFs induce an upregulation of cholesterol biosynthesis genes (3-hydroxy-3-methylglutaryl-CoA synthase 2 (*HMGCS2), DHCR7*, 24-dehydrocholesterol reductase *(DHCR24)*, methylsterol monooxygenase 1 *(SC4MOL)* and sterol-C5-desaturase *(SC5DL)*). Additionally, sterol biosynthesis genes (aldo-keto reductase family 1 member C3 (*AKR1C3)*, aldo-keto reductase family 1 member C4 *(AKR1C4)*, UDP-glucuronosyltransferase family 1 member A1 *(UGT1A1)*, UDP-glucuronosyltransferase family 2 member B7 *(UGT2B7)*, UDP-glucuronosyltransferase family 2 member B10 *(UGT2B10)*, UDP-glucuronosyltransferase family 2 member B17 *(UGT2B17)*) are also upregulated to mediate androgen receptor targeted therapy resistance (102). Importantly, knockdown of *HMGCS2* significantly impaired 3D spheroid growth whereas ectopic expression of *HMGCS2* significantly increased LNCaP prostate cancer spheroid growth.

Seeing that the human brain contains the highest level of cholesterol in the body (103), it is not surprising to observe several studies implicating cholesterol in the maintenance of stemness and malignant potential. Shakya and colleagues employed spatial capture ribonucleic acid (RNA)-sequencing revealing several differences in lipid-related gene expression with increased expression of cholesterol homeostasis genes in glioblastoma (GBM) organoid core when compared to the cell population at the rim (104). Furthermore, the hypoxic organoid core displayed an accumulation of lipid droplets when compared to the rim. Further investigation by this group revealed that CSCs are enriched among cells with decreased lipid droplet accumulation, which occurs at the rim, and the accumulation of lipid droplets could be utilized as a means to classify the stemness state of glioblastoma cells (104). This can be attributed to the efficiency of GBM organoids in mimicking the perivascular niche (high oxygen, high nutrient content) where CSCs are typically found. In support of this study, Lewis et al. (105), identified a crucial role for SREBP1 in maintaining glioblastoma viability under oxygen and lipid-deplete conditions, where silencing of SREBP1 led to a significant reduction in spheroid growth (105). Furthermore, SREBP1 defines a gene signature that is associated with poor prognosis of glioblastoma multiforme. This is evidenced by SREBPs ability to regulate several cancer-related genes including genes involved in cholesterol and fatty acid metabolism, inflammation, cancer stem cell signalling, chemotaxis as well as oxidative stress (105). Studies conducted in glioblastoma also document increased levels of cholesterol in patient-derived spheroid cultures with increased gene expression noted in *FDFT1, FDPS*, and *HMGCS1* relative to their differentiated counterparts (106). Further analysis highlighted the importance of *FDPS* in maintaining stemness of glioblastoma cells where knockdown or pharmacological inhibition of *FDPS* inhibited stemness and sphere formation efficiency (106). Similarly, extracranial tumours such as neuroblastomas display an inherent reliance on cholesterol for maintaining neuroblastoma sphere-forming cells (107). Sterol regulatory element-binding protein 2 (SREBF2) mediated a significant upregulation of *HMGCS1, HMGCR, MVK*, phosphoglycerate dehydrogenase (*PHGDH)*, phosphoserine aminotransferase 1 *(PSAT1)*, phosphoserine phosphatase *(PSPH)*, and serine hydroxymethyltransferase 2 (*SHMT2)* and consequently served as crucial transcriptional regulator in these cells (107). Importantly, increased expression of cholesterol-synthesis related genes is prevalent in individuals diagnosed with high-risk neoblastomas, and is significantly associated with a poor clinical outcome (107).

From the above studies it can be stated that the deregulation of crucial cholesterol related genes including *HMGCS1, FDFT1* and *FDPS* may serve as a common transcriptomic signature in the most common cancer types including breast, colorectal and brain. These genes should be prioritized in further studies delineating the role of cholesterol in cancer in several other cancer types that are yet to be investigated. It is important to emphasize, *HMGCS1, FDFT1* and *FDPS* are commonly involved in cholesterol biosynthesis hence potentiating the use of therapeutics that target cholesterol biosynthesis as an effective therapeutic across several cancer types. In this light, studies conducted attempting to target cellular cholesterol in 3D models will be further elaborated on below.

### 4.3 Targeting cholesterol as an anti-cancer therapeutic in 3D cultures

Attempts at targeting aberrant cholesterol metabolism is an actively explored field in cancer. The most well elucidated means of achieving this is by employing a class of cholesterol synthesis inhibitors termed statins. These drugs serve as competitive inhibitors of HMGCR, by directly blocking the enzyme’s active site and impeding the conversion of HMG-CoA to mevalonate, which serves as the proximal and rate-limiting step of cholesterol biosynthesis (108). On this basis, evidence exists documenting therapeutic benefits associated with targeting cholesterol in CRC-derived spheroids. Studies conducted by Zhang et al. (109), document that treating CRC-derived spheroids with pitavastatin led to a dose-dependent reduction in spheroid growth (109). Furthermore, pitavastatin treatment reduced the expression of the multidrug resistance gene 1 (MDR1) protein in CSCs and further decreased the expression of CSC-related genes including octamer-binding transcription factor 4 (*OCT4)*, sex determining region Y-box 2 *(SOX2)* and NK2-family homeobox transcription factor (*NANOG)* (109). Interestingly, recent evidence documents that targeting cholesterol through statin treatment facilitates the loss of stem cells and leads to the induction of differentiation in CRC organoids. Statins were seen to increase the secretory lineages such as the enteroendocrine and paneth cells and decrease the stem cell frequency. Additionally, many of the other identified drugs exhibited a mechanism of action targeting the cholesterol pathway and displayed a preferential therapeutic benefit by acting selectively on cancer and not wild type organoids (110). Authors hence postulate that differentiation induction may contribute crucially to determining drug efficacy. These findings can be further supported by studies conducted in prostate cancer, where administering simvastatin to cancer spheroids significantly impeded the growth of castration and enzalutamide resistant cells (102). Similarly, studies conducted by Deezagi and Safari (78), highlight therapeutic benefits associated with rosuvastatin administration in prostate cancer spheroids demonstrating decreased proliferation and spheroid formation efficiency following treatment with rosuvastatin (78).

Importantly, the targeting of cholesterol can also prove beneficial in restoring sensitivity to conventional therapeutics or in the event of synthetic lethality resulting in anti-tumour synergy. This is evidenced by Gao and colleagues as they document synergistic effects observed when a cholesterol targeting agent (lovastatin or zoledronate acid) is combined with a chemotherapeutic (5-fluorouracil) (100). Similarly in gastric cancer, combining docetaxel with lovastatin proved efficient in inhibiting spheroid growth and inducing apoptosis (111). Focusing on breast cancer organoids, studies implicate the nuclear receptor RAR-related orphan receptor gamma (ROR-γ) as a crucial activator of the cholesterol biosynthesis pathway by mediating SREBP2 chromatin recruitment and subsequent activation (112). Consequently, ROR-γ inhibitors in combination with statins synergistically target TNBC cells resulting in tumor regression and suppression of metastasis. Importantly, these inhibitors preserve host cholesterol homeostasis making it an attractive targeted therapy for TNBC patients (112). Furthermore, there are beneficial effects of administering cyclodextrin nanocarriers to enhance the anticancer efficacy of erlotinib (commonly prescribed for small cell lung cancer). (113). This can be justified by cholesterols regulatory role on membrane cholesterol as well as the functionality of key drug efflux pumps. Hence, it can be educed that cholesterol biosynthesis could serve as a potential genetic vulnerability in cancer, potentiating the combination of cholesterol-targeting agents with other therapies.

A more recently developed approach of targeting cholesterol in cancer includes targeting the uptake of cholesterol in cancers that display an inherent reliance on LDLR-derived cholesterol. One such means of achieving this is by employing LDLR-targeting peptides. *In silico* and *in vitro* work in spheroids illustrate abundant LDLR expression in the epithelial compartment of patient PDAC irrespective of tumor size, stage or aggressiveness (114). Importantly, treatment with LDLR-targeting peptide FC (A680) VH417 resulted in selective targeting of PDAC tissue with high levels of desmoplasia. Furthermore, this peptide effectively distinguished chronic pancreatitis from pancreatic tumor eliminating hepto- and nephro-toxicity (114). Seeing that these peptides mimic the uptake process of LDL (LDLR- mediated endocytosis and transfer to the late endosomal/lysosomal compartment), this can be exploited to deliver anticancer molecules to lysosomes resulting in targeted drug delivery (114). In a different study, Lee et al. (2022) implicate the LDL-targeting drug lomitapide in facilitating hyperactivation of autophagy which suppresses tumor growth and increases cancer cell death in CRC organoids (115). This was attributed to direct inhibition of mammalian target of rapamycin (mTOR) kinase activity and consequent regulation of mTOR signaling. Interestingly lomitapide proved more effective than 5-fluorouracil in decreasing organoid viability hence potentiating the use of lomitapide as anticancer therapeutic in CRC (115).

Several of the summarised studies attempt to shed light on the dynamics of targeting cholesterol in 3D cultures. To date studies conducted have mainly focused on cholesterol synthesis inhibitors and LDL-targeting agents to reduce cellular cholesterol content resulting in reduced growth and stemness potential. Importantly, combining statin treatment with conventional therapies results in anti-tumor synergy potentiating the use of cholesterol targeting agents either as single or dual therapeutics in an attempt to combat tumorigenesis. Despite these positive results, treatment with statins has documented several side effects in the clinical setting, highlighting the incipient need to explore alternative means of targeting cellular cholesterol. One such way of achieving this is by employing cholesterol depletory agents termed cyclodextrins, an area our laboratory actively focuses on. Cyclodextrins have been used in 2D *in-vitro* and *in-vivo* studies depleting excess plasma membrane and lipid raft cholesterol from cancer cells. The decrease in membrane cholesterol leads to lipid raft disruption, which consequently affects cell signaling intermediates that cells subvert to promote tumorigenesis (116). Additionally, a decrease in membrane cholesterol levels is associated with increased drug permeability and consequently enhanced sensitivity in cancer cells (116, 117). On this basis, exploring this research area will provide crucial information pertaining to whether membrane cholesterol depletion rather than cholesterol synthesis inhibition could serve as a better approach to combat cancer progression.

Considering the pivotal role of cholesterol in cancer, the final subsection of the review focuses on some interesting insights obtained from existing disease models elucidating the importance of cholesterol in supporting cancer initiation and progression.

### 4.4 Relevance of 3D models to delineate the role of cholesterol in disease pathogenesis

Classical cell lines and animal model systems have served as the major driving force of cancer research up until the early twenty-first century. Despite the advantages of this culturing technique several limitations exist, resulting in the inception of 3D culturing techniques as a more physiologically relevant disease model. Following the successful establishment of human organoid systems, efforts are currently underway to model several relevant diseases with therapeutic intent. In the following section, we have attempted to summarize studies conducted implicating cholesterol in cancer pathogenesis.

The link between dietary cholesterol and cancer has been well established. While the association does vary between cancer types, in many cancer types excess dietary consumption of cholesterol is associated with cancer risk, one such cancer being CRC (118). Consequently, this has been exploited to identify a possible mechanism indicating how obesity drives CRC progression in mouse organoid models (119). Deregulations to the Wnt signaling pathway has been linked with an increased risk of developing obesity (120). In this study, administration of a small molecule, adiponectin receptor agonist (AdipoRon), was seen to attenuate Wnt signaling by decreasing the plasma membrane rigidity. Importantly, the Wnt signaling pathway is crucial to CRC onset. The decrease in plasma membrane rigidity corroborated with a decrease in plasma membrane free cholesterol and intracellular accumulation of cholesterol in lysosomes (119). This suggests that an aberrant Wnt signaling pathway in the highly obese population could thus be targeted as a potential means to prevent obesity-induced tumorigenesis.

In addition to modelling cancer initiation, the role of cholesterol in cancer progression has also been delineated through the use of organoid cultures. Work conducted in pancreatic cancer organoids provides evidence for a potential role of cholesterol in facilitating acinar to ductal metaplasia (121). Authors document increased production of acetyl-CoA in KRAS^G12D^ mutant acinar cells. Interestingly, ATP citrate lyase (ACLY) mediated production of acetyl-CoA mediates increased production of HMG-CoA thereby promoting acinar to ductal metaplasia (121). Consequently, depletion of ACLY decreased the production of acetyl-CoA and HMG-CoA, which impeded duct formation and preserved acinar morphology. *In vivo* this translated to decreased tumor burden and improved survival (121). On this basis, KRAS-driven metabolic alterations can be exploited as potential therapy in pancreatic cancer.

Interestingly, organoids have recently proved useful in studying interactions between human systems and microorganisms resulting in novel insights into the role of the microbiome in cancer etiology. Bacteria have the capability to manipulate cellular cholesterol levels to mediate carcinogenesis. Cholesterol can act as a mediator of inflammation in response to *Helicobacter pylori* (*H.pylori*) infection. Mechanistic investigations indicate that disruption of lipid rafts by *H.pylori* is attributed to the enzyme cholesterol-α-glucosyltransferase (122). Depletion of cholesterol impedes the assembly of interferon (IFN)-gamma (γ) receptors and further abrogates activation of the janus kinase/signal transducers and activators of transcription 1 (JAK/STAT1) signaling pathway in human organoids (122). Furthermore, the *H. pylori* mediated cholesterol depletion in addition to impeding the IFN-γ further inhibits the Type I IFN-response, interleukin (IL)-6 and IL-22. This consequently allows bacteria to escape the host inflammatory response and could serve as a potential mechanism whereby *H. pylori* mediates gastric carcinogenesis (122). This is consistent with evidence that patients that suffer with increased blood cholesterol often exhibit severe *H. pylori*-induced gastritis, resulting in inflammation of the gastrointestinal tract and may increase the risk of gastric cancer (122, 123). In another study conducted, beyond its direct oncogenic effects, SQLE has also shown to impact the gut microbiota leading to gut dysbiosis. This consequently leads to gut-barrier dysfunction promoting a pro-inflammatory state in the colon which further facilitated colon proliferation (124). Importantly, treating CRC cell lines and organoids with a SQLE inhibitor, terbinafine, in combination with either 5-fluorouracil or oxaliplatin potentiated a synergistic effect in impairing CRC carcinogenesis. This study further validates the importance of targeting SQLE as a potential means to improve chemotherapeutic efficacy in CRC (C. 124).

**Figure 4:**
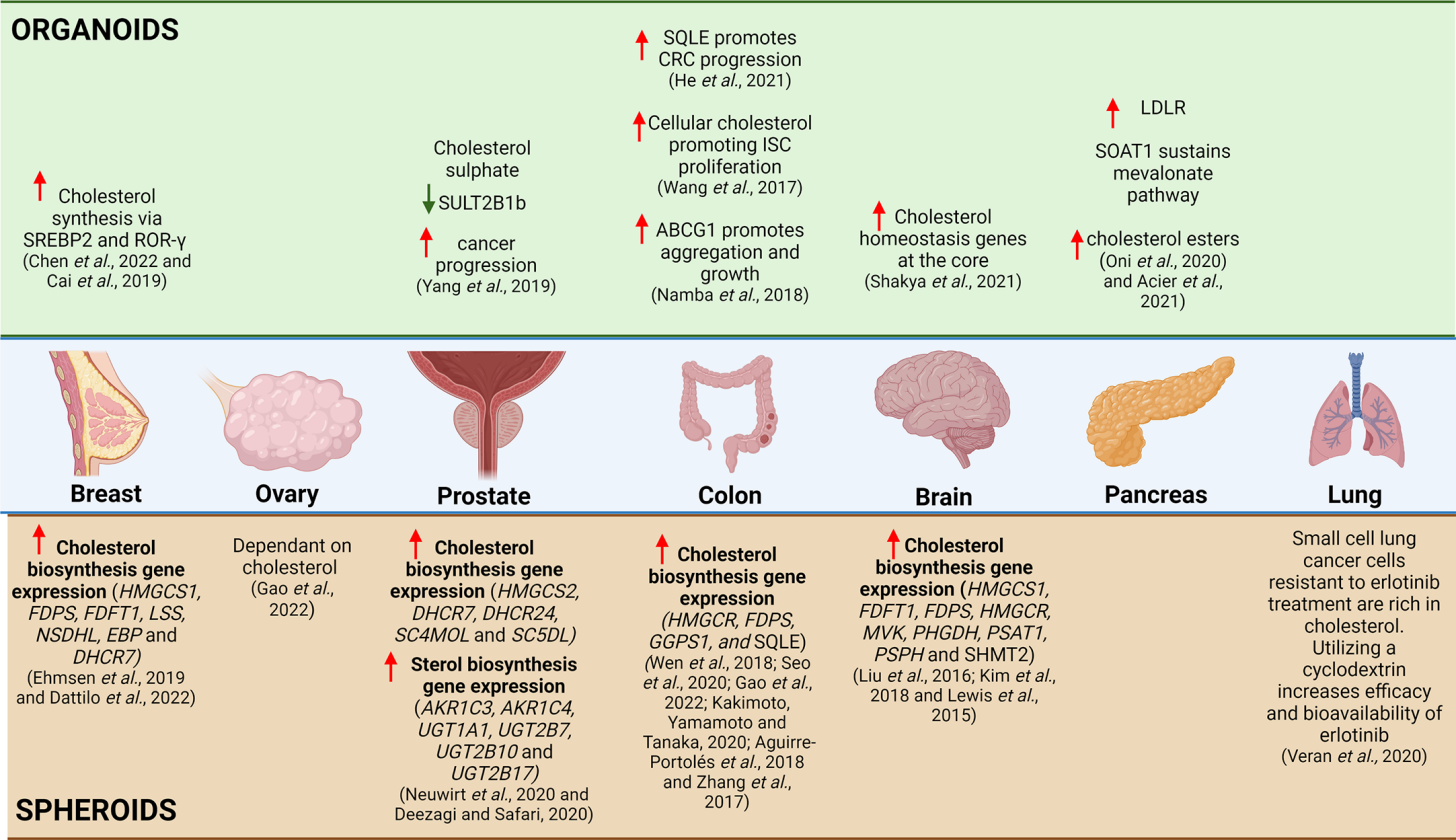
Summary of Cholesterol Related Research Utilizing 3D *in-vitro* Models. Based on literature, studies investigating the relationship between breast cancer and cholesterol, it was evident that increased cholesterol synthesis occurs via SREBP2 in organoids and that there is increased cholesterol biosynthesis gene expression in spheroids. The relationship between cholesterol synthesis and ovarian cancer has yet to be explored in organoids, but in a small study utilizing spheroids, it was shown that the ovarian cancer spheroids are dependent on cholesterol. In prostate cancer, the utilization of organoids has illustrated that cholesterol sulphate decreases SULT2B1b, which correlates to increased cancer progression and in spheroids it has illustrated increased expression of cholesterol biosynthesis genes and sterol biosynthesis genes. The use of intestinal organoids has shown that SQLE increases CRC progression, increased cellular cholesterol promoting ISC proliferation and increased ABCG1 promotes aggregation and growth of intestinal cancer cells, whereas spheroids have illustrated increased expression of cholesterol biosynthesis genes. In glioblastoma, the use of spheroids have illustrated increased expression of cholesterol biosynthesis genes and in organoids it was shown that there was increased expression of cholesterol homeostasis genes at the organoid core. In pancreatic cancer, organoids have been used to illustrate increased LDLR, SOAT1 sustains the mevalonate pathway and there is increased cholesterol esters present. In lung cancer, there are limited studies that explore the link between cholesterol and cancer, but a small study has illustrated that utilizing a cyclodextrin as a drug vehicle for delivery in cholesterol-rich erlotinib resistant small cell lung cancer cells can be a potentially useful therapeutic mechanism.

Based on the above studies (**Figure 4**) it is evident that the inception of 3D culture systems potentiates the use of these systems in understanding and delineating the complexities pertaining to cholesterol metabolism in cancer and serves as a more advanced model of disease modeling and anticancer screening. Numerous studies have highlighted the potential of organoids in biobanking, anticancer drug screening and the identification of prognostic biomarkers (12, 16, 125). The utilization of organoids may also be perceived as more advantageous in comparison to spheroids as relatively small amounts of tissue are required and are easily grown from patients from different regions of the patients’ tumor recapitulating tissue heterogeneity (16). While several crucial insights have been obtained thus far, further attempts must be enforced to study additional cancer types and to further elucidate the multifaceted role of cholesterol in cancer. This can be achieved by investigating the influence of cholesterol on supporting cellular and non-cellular components of the tumor microenvironment (TME) through co-culturing. Consequently, completing these studies will facilitate a wholistic view on the role of supporting cells in the tumor microenvironment and how they influence cholesterol metabolism to facilitate disease progression. Importantly, many studies are to be expected in the near future as this model becomes more widely used. Consequently, this provides a more efficient platform for the inception of personalized medicine approaches in a cancer setting.

## 5 Conclusions and future perspectives

Cholesterol is a crucial lipid known to maintain cellular homeostasis by regulating the survival and growth of cells. The abundance of cholesterol present in cancerous cells allows for not only increased growth and proliferation but maintains lipid raft integrity, which is crucial for numerous signaling pathways that dictate growth, proliferation, metastasis, and drug resistance. While several studies have been conducted elucidating the molecular mechanisms of cholesterol homeostasis, this has been predominantly explored in 2D cell line and mouse xenograft models which display inherent limitations.

To combat this, recent scientific advances elucidate the benefits associated with culturing cells in 3D systems as opposed to 2D models. The well explored 3D models investigated include spheroid and organoid cultures. Spheroids are derived from cell lines and can include mixtures of cell types and primary cells (16). Culturing spheroids obtains organ physiology as it is layers of heterogeneous cells, which briefly resembles 3D cellular organization (16). While spheroid cultures are derived from cell lines, organoid cultures are derived from human tissue and serve as a superior model to both 2D cell lines and spheroid cultures. The utility of organoids to studying disease progression and treatment is endless and have been increasingly employed in either basic or translational research. Despite the use of organoids in studying cancer biology, the novel applications of organoids are the utilization of lung organoids for lung disease that may be transplanted in these individuals instead of undergoing a lung transplant and the generation of vascularized brain organoids that can be utilized to study neurovascular interaction (126, 127). Additionally, the generation of skin organoids that have similar complexity of the skin (including the development of hair follicles) and eye organoids that could potentially be used to replace neurons lost in humans due to degenerative eye diseases, therefore providing a potential therapeutic option (128, 129). Furthermore, organoids can be stably cultured long-term, have proved useful in disease modelling, maintaining genetic and phenotypic heterogeneity and can elucidate inter- and intra-tumor heterogeneity. For this reason, they serve as a promising tool for personalized medicine and clinical applications.

From a 3D culture perspective, emerging evidence documenting the indispensable role of cholesterol as well as its metabolites in mediating cancer progression, development and drug resistance becomes known. By culturing organoids in 3D, this allows for mimicking oxygen, nutrient and drug exposure consequently ensuring an accurate physiological environment is maintained. This allows more informed therapeutic outcomes to be attained. In this light, several studies support the hypothesis that cholesterol metabolism could serve as a potential genetic vulnerability in 3D cultures, thereby potentiating the combination of cholesterol-targeting agents with chemotherapeutic agents. Furthermore, the use of these culture types allows for the identification of subtype-specific as well as a tissue-specific response to therapeutics. While the most well-explored cholesterol targeting agents are statins, these function as cholesterol synthesis inhibitors and have debilitating side effects on healthy cells. To combat this, novel cholesterol targeting agents should be explored.

Moreover, evidence exists implicating cholesterol and its metabolites in influencing components of the TME. In these supporting cells, the level of cholesterol is finely balanced with any perturbations proving detrimental to supporting cells of the TME. Consequently, the ability of organoids to be co-cultured with additional components from the microenvironment including fibroblasts, immune cells and endothelial cells allows to manipulate both cellular as well as tumor microenvironmental levels of cholesterol. The development of such models would assist in understanding the multifaceted role that cholesterol plays in the TME. Additionally, organoids can be co-cultured with microorganisms and the role of cholesterol in facilitating microbial infections and disease should also be further explored.

It is important to note, despite the potential use and applications of 3D culture, there are a number of considerations and limitations that would need to be overcome. One of the major limitations is the cost of 3D culture, which is exponentially more expensive than that of 2D culture (58). Hopefully, as 3D culture becomes more widely used and resources become more easily available the cost of this culture method would decrease thus allowing many more labs especially from the developing world to contribute effectively to substantiating 3D models as go to methods for progressing our understanding of disease biology. Another major limitation is the type of basement membrane used to mimic the ECM is derived from animal cells or is synthetically produced (75). The composition of the basement membrane extracts is undefined and there tends to be variations between batches, which can result in experimental variation (60, 130). This can ultimately affect the reproducibility of results that are generated. It is also important to note, that the chemical and physical properties of the ECM mimic used can interfere with downstream experiments, such as RNA extraction which can present with protein contamination from the ECM mimic (75). Furthermore, it can be difficult to recapitulate cell microenvironments and the addition of co-culturing adds an additional layer of complexity to an already complex culture method (58). Attempting to co-culture in addition to performing 3D culture requires rigorous optimization as cell ratios, cell growth rates and culture media requirements need to be considered in order to obtain functional tissues (75). An additional issue that arises is that cells isolated from normal tissue could potentially overgrow tumoral cells present in cancerous tissue (131). Hence it is of importance to ensure complete isolation of the correct type of cells (normal or tumoral cells) prior to culture and to characterize the cells being grown in-depth (131). Therefore, 3D culture models may serve as a good alternative to 2D cell culture, but it is important to understand the nature of the experiments performed as well as all chemical and physical properties that may influence the results generated. The culture media used between 2D and 3D cell culture is quite different, in that 3D culture media contains more growth factors and reagents required to ensure growth of specific cell types and stem cells. The more general culture media used for 2D cell culture contain sufficient nutrients for growth and can also be adjusted to include the required growth factors and reagents to support 3D culture. However, the culture media used will affect metabolism and cell growth, which may impact cholesterol metabolism and may be interesting to explore further (132).

In conclusion, there is strong evidence documenting the multi-faceted role of cholesterol in cancer. While a few studies have utilized 3D culture models to study the complex relationship between cancer and cholesterol, well-established links across cancer types and comparisons within 3D models are lacking. Additionally, limited studies are available delineating the role of cholesterol in the TME. Given that cancer cells express higher levels of cholesterol and the significance of cholesterol dyshomeostasis during initiation and progression of cancer, understanding the role of cholesterol in more complex culture methods may prove to be advantageous in justifying whether cholesterol could serve as a therapeutic vulnerability in cancer. Therefore, organoid cultures present a unique opportunity to investigate complex cellular pathways and interactions, with a potential to facilitate patient-centric translational benefits utilizing laboratory-based research.

## Author contributions

T-LDP and NA conceived, designed, and wrote the review. All authors contributed to the article and approved the submitted version. T-LDP designed figures. MK contributed to the conceptualization, figures, editing, and the final review. MK also provided funding and supervision.

## References

[1] Bray F Ferlay J Soerjomataram I Siegel RL Torre LA Jemal A. Global cancer statistics 2018: GLOBOCAN estimates of incidence and mortality worldwide for 36 cancers in 185 countries. CA: A Cancer J Clin (2018) 68(6):394–4245. doi: 10.3322/caac.21492 30207593

[2] Sung H Ferlay J Siegel RL Laversanne M Soerjomataram I Jemal A. Global cancer statistics 2020: GLOBOCAN estimates of incidence and mortality worldwide for 36 cancers in 185 countries. CA: A Cancer J Clin (2021) 71(3):1–415. doi: 10.3322/caac.21660 33538338

[3] Hanahan D Weinberg RA. Biological hallmarks of cancer. Holland-Frei Cancer Med (2017) 2008. doi: 10.1002/9781119000822.hfcm002

[4] Hanahan D. Hallmarks of cancer: New dimensions. Cancer Discovery (2022) 12(1):31–46. doi: 10.1158/2159-8290.CD-21-1059 35022204

[5] Cheng H Wang M Su J Li Y Long J Chu J. Lipid metabolism and cancer. Life (2022) 12:784. doi: 10.3390/life12060784 35743814 PMC9224822

[6] Vasan N Baselga J Hyman DM. A view on drug resistance in cancer. Nature (2019) 575(7782):299–309. doi: 10.1038/s41586-019-1730-1 31723286 PMC8008476

[7] Mansoori B Mohammadi A Davudian S Shirjang S Baradaran B. The different mechanisms of cancer drug resistance: A brief review. Adv Pharm Bull (2017) 7(3):339–485. doi: 10.15171/apb.2017.041 29071215 PMC5651054

[8] Altea-Manzano P Cuadros AM Broadfield LA Fendt S-M. Nutrient metabolism and cancer in the in vivo context: A metabolic game of give and take. EMBO Rep (2020) 21(10):e50635. doi: 10.15252/embr.202050635 32964587 PMC7534637

[9] Broadfield LA Pane AA Talebi A Swinnen JV Fendt SM. Lipid metabolism in cancer: New perspectives and emerging mechanisms. Dev Cell (2021) 56(10):1363–93. doi: 10.1016/j.devcel.2021.04.013 33945792

[10] Giacomini I Gianfanti F Desbats MA Orso G Berretta M Prayer-Galetti T. Cholesterol metabolic reprogramming in cancer and its pharmacological modulation as therapeutic strategy. Front Oncol (2021) 11:682911. doi: 10.3389/fonc.2021.682911 34109128 PMC8181394

[11] Gu L Saha ST Thomas J Kaur M. Targeting cellular cholesterol for anticancer therapy. FEBS J (2019) 286(21):4192–208. doi: 10.1111/febs.15018 31350867

[12] Yan HHN Siu HC Law S Ho SL Yue SSK Tsui WY. A comprehensive human gastric cancer organoid biobank captures tumor subtype heterogeneity and enables therapeutic screening. Cell Stem Cell (2018) 23(6):882–97.e11. doi: 10.1016/j.stem.2018.09.016 30344100

[13] D’Costa K Kosic M Lam A Moradipour A Zhao Y Radisic M. Biomaterials and culture systems for development of organoid and organ-on-a-Chip models. Ann Biomed Eng (2020) 48(7):2002–27. doi: 10.1007/s10439-020-02498-w PMC7334104 32285341

[14] Sajjad H Imtiaz S Noor T Siddiqui YH Sajjad A Zia M. Cancer models in preclinical research: A chronicle review of advancement in effective cancer research. Anim Models Exp Med (2021) 4(2):87–103. doi: 10.1002/ame2.12165 PMC8212826 34179717

[15] Franchi-Mendes T Eduardo R Domenici G Brito C. 3D cancer models: Depicting cellular crosstalk within the tumour microenvironment. Cancers (2021) 13(18):4610. doi: 10.3390/cancers13184610 34572836 PMC8468887

[16] Gunti S Hoke ATK Vu KP London NR. Organoid and spheroid tumor models: Techniques and applications. Cancers (2021) 13(4):874. doi: 10.3390/cancers13040874 33669619 PMC7922036

[17] Sato T Vries RG Snippert HJ van de Wetering M Barker N Daniel E. Single Lgr5 stem cells build crypt-villus structures *in vitro* without a mesenchymal niche. Nature (2009) 459(7244):262–65. doi: 10.1038/nature07935 19329995

[18] Tuveson D Clevers H. Cancer modeling meets human organoid technology. Science (2019) 364(6444):952–5. doi: 10.1126/science.aaw6985 31171691

[19] Mc Auley MT Wilkinson DJ Jones JJL Kirkwood TBL. A whole-body mathematical model of cholesterol metabolism and its age-associated dysregulation. BMC Syst Biol (2012) 6(1):130. doi: 10.1186/1752-0509-6-130 23046614 PMC3574035

[20] Luo J Yang H Song BL. Mechanisms and regulation of cholesterol homeostasis. Nat Rev Mol Cell Biol (2020) 21(4):225–45. doi: 10.1038/s41580-019-0190-7 31848472

[21] Kuzu OF Noory MA Robertson GP. The role of cholesterol in cancer. Cancer Res (2016) 76(8):2063–70. doi: 10.1158/0008-5472.CAN-15-2613 PMC5813477 27197250

[22] Zhang Y Zhang J Li Q Wu Y Wang D Xu L. Cholesterol content in cell membrane maintains surface levels of ErbB2 and confers a therapeutic vulnerability in ErbB2-positive breast cancer. Cell Commun Signaling (2019) 17(1):15. doi: 10.1186/s12964-019-0328-4 PMC6383291 30786890

[23] Abdulla Naaziyah C Vincent T Mandeep K. Mechanistic insights delineating the role of cholesterol in epithelial mesenchymal transition and drug resistance in cancer. Front Cell Dev Biol (2021) 9:728325. doi: 10.3389/fcell.2021.728325 34869315 PMC8640133

[24] Moon H Ruelcke JE Choi E Sharpe LJ Nassar ZD Bielefeldt-Ohmann H. Diet-induced hypercholesterolemia promotes androgen-independent prostate cancer metastasis *via* IQGAP1 and caveolin-1. Oncotarget (2015) 6(10):7438–53. doi: 10.18632/oncotarget.3476 PMC4480691 25924234

[25] Du Q Wang Q Fan H Wang J Liu X Wang H. Dietary cholesterol promotes AOM-induced colorectal cancer through activating the NLRP3 inflammasome. Biochem Pharmacol (2016) 105:42–54. doi: 10.1016/j.bcp.2016.02.017 26921636

[26] Ding X Zhang W Li S Yang H. The role of cholesterol metabolism in cancer. Am J Cancer Res (2019) 9(2):219–75. PMC6405981 30906624

[27] Gonzalez-Ortiz A Galindo-Hernandez O Hernandez-Acevedo GN Hurtado-Ureta G Garcia-Gonzalez V. Impact of cholesterol-pathways on breast cancer development, a metabolic landscape. J Cancer (2021) 12(14):4307–21. doi: 10.7150/jca.54637 PMC8176427 34093831

[28] Murai T. Cholesterol lowering: Role in cancer prevention and treatment. Biol Chem (2015) 396(1):1–11. doi: 10.1515/hsz-2014-0194 25205720

[29] Jiang T Zhang G Lou Z. Role of the sterol regulatory element binding protein pathway in tumorigenesis. Front Oncol (2020) 10:1788. doi: 10.3389/fonc.2020.01788 33014877 PMC7506081

[30] Duan Y Gong K Xu S Zhang F Meng X Han J. Regulation of cholesterol homeostasis in health and diseases: From mechanisms to targeted therapeutics. Signal Transduct Targeted Ther (2022) 7(1):265. doi: 10.1038/s41392-022-01125-5 PMC9344793 35918332

[31] Mok EHoK Wah Lee TK. The pivotal role of the dysregulation of cholesterol homeostasis in cancer: Implications for therapeutic targets. Cancers 2020 (2020) 12(6):14105. doi: 10.3390/CANCERS12061410 PMC7352357 32486083

[32] Peterson AS Fong LG Young SG. PCSK9 function and physiology. J Lipid Res (2008) 49(6):1152–6. doi: 10.1194/jlr.E800008-JLR200 PMC2386899 18375913

[33] Mahboobnia K Pirro M Marini E Grignani F Bezsonov EE Jamialahmadi T. PCSK9 and cancer: Rethinking the link. Biomedicine Pharmacother (2021) 140:111758. doi: 10.1016/j.biopha.2021.111758 34058443

[34] Li J Gu D Lee SSY Song B Bandyopadhyay S Chen S. Abrogating cholesterol esterification suppresses growth and metastasis of pancreatic cancer. Oncogene (2016) 35(50):6378–88. doi: 10.1038/onc.2016.168 PMC5093084 27132508

[35] Vona R Iessi E Matarrese P. Role of cholesterol and lipid rafts in cancer signaling: A promising therapeutic opportunity? Front Cell Dev Biol (2021) 9:622908. doi: 10.3389/fcell.2021.622908 33816471 PMC8017202

[36] Li J Qu X Tian J Zhang JT Cheng JiX. Cholesterol esterification inhibition and gemcitabine synergistically suppress pancreatic ductal adenocarcinoma proliferation. PloS One (2018) 13(2):e0193318. doi: 10.1371/journal.pone.0193318 29489864 PMC5831104

[37] Zabielska J Sledzinski T Stelmanska E. Acyl-coenzyme a: Cholesterol acyltransferase inhibition in cancer treatment. Anticancer Res (2019) 39(7):3385–94. doi: 10.21873/anticanres.13482 31262860

[38] Esau L Sagar S Bangarusamy D Kaur M. Identification of CETP as a molecular target for estrogen positive breast cancer cell death by cholesterol depleting agents. Genes Cancer (2016) 7(9–10):309–22. doi: 10.18632/genesandcancer.122 PMC5115172 28050232

[39] de Gonzalo-Calvo D López-Vilaró L Nasarre L Perez-Olabarria M Vázquez T Escuin D. Intratumor cholesteryl ester accumulation is associated with human breast cancer proliferation and aggressive potential: A molecular and clinicopathological study. BMC Cancer (2015) 15(1):460. doi: 10.1186/s12885-015-1469-5 26055977 PMC4460760

[40] Mollinedo F Gajate C. Lipid rafts as signaling hubs in cancer cell Survival/Death and invasion: Implications in tumor progression and therapy. J Lipid Res (2020) 61(5):611–35. doi: 10.1194/jlr.TR119000439 33715811 PMC7193951

[41] Yan A Jia Z Qiao C Wang M Ding X. Cholesterol metabolism in drug−resistant cancer (Review). Int J Oncol (2020) 57(5):1103–55. doi: 10.3892/ijo.2020.5124 33491740

[42] Wu Y Si R Tang H He Z Zhu H Wang L. Cholesterol reduces the sensitivity to platinum-based chemotherapy via upregulating ABCG2 in lung adenocarcinoma. Biochem Biophys Res Commun (2015) 457(4):614–20. doi: 10.1016/J.BBRC.2015.01.035 25603057

[43] Zhang P Wang D Zhao Y Ren S Gao K Ye Z. Intrinsic BET inhibitor resistance in prostate cancer caused by SPOP mutation-mediated BET protein stabilization. Physiol Behav (2015) 23(9):1055–62. doi: 10.1038/nm.4379.Intrinsic PMC5653288 28805822

[44] Murtola TJ Peltomaa AI Talala K Määttänen L Taari K Tammela TLJ. Statin use and prostate cancer survival in the Finnish randomized study of screening for prostate cancer. Eur Urol Focus (2017) 3(2–3):212–20. doi: 10.1016/j.euf.2016.05.004 28753762

[45] Sperling CD Verdoodt F Hansen MK Dehlendorff C Friis S Kjaer SK. Statin use and mortality among endometrial cancer patients: A Danish nationwide cohort study. Int J Cancer (2018) 143(11):2668–76. doi: 10.1002/ijc.31625 29923185

[46] Kopecka J Trouillas P Gašparović AČ Gazzano E Assaraf YG Rigante C. Phospholipids and cholesterol: Inducers of cancer multidrug resistance and therapeutic targets. Drug Resist Updat (2020) 49:100670. doi: 10.1016/j.drup.2019.100670 31846838

[47] Zalba S ten Hagen TLM. Cell membrane modulation as adjuvant in cancer therapy. Cancer Treat Rev (2017) 52:48–57. doi: 10.1016/j.ctrv.2016.10.008 PMC5195909 27889637

[48] Białkowska K Komorowski P Bryszewska M Miłowska K. Spheroids as a type of three-dimensional cell cultures–examples of methods of preparation and the most important application. Int J Mol Sci (2020) 21(17):6225. doi: 10.3390/ijms21176225 32872135 PMC7503223

[49] Pape J Emberton M Cheema U. 3D cancer models: The need for a complex stroma, compartmentalization and stiffness. Front Bioeng Biotechnol (2021) 9:660502. doi: 10.3389/fbioe.2021.660502 33912551 PMC8072339

[50] Habanjar O Diab-Assaf M Caldefie-Chezet F Delort. L. 3D cell culture systems: Tumor application, advantages, and disadvantages. Int J Mol Sci (2021) 22(22):12200. doi: 10.3390/ijms222212200 34830082 PMC8618305

[51] Nicolas J Magli S Rabbachin L Sampaolesi S Nicotra F Russo L. 3D extracellular matrix mimics: Fundamental concepts and role of materials chemistry to influence stem cell fate. Biomacromolecules (2020) 21(6):1968–94. doi: 10.1021/acs.biomac.0c00045 32227919

[52] Yang H Sun L Liu M Mao Y. Patient-derived organoids: A promising model for personalized cancer treatment. Gastroenterol Rep (2018) 6(4):243–45. doi: 10.1093/gastro/goy040 PMC6225812 30430011

[53] Clevers H. Modeling development and disease with organoids. Cell (2016) 165(7):1586–97. doi: 10.1016/j.cell.2016.05.082 27315476

[54] Boonekamp KE Dayton TL Clevers H. Intestinal organoids as tools for enriching and studying specific and rare cell types: Advances and future directions. J Mol Cell Biol (2020) 12(8):562–68. doi: 10.1093/jmcb/mjaa034 32667995 PMC7683021

[55] Rizzo G Bertotti A Leto SM Vetrano S. Patient-derived tumor models: A more suitable tool for pre-clinical studies in colorectal cancer. J Exp Clin Cancer Res (2021) 40(1):178. doi: 10.1186/s13046-021-01970-2 34074330 PMC8168319

[56] Kim J Koo BK Knoblich JA. Human organoids: Model systems for human biology and medicine. Nat Rev Mol Cell Biol (2020) 21(10):571–84. doi: 10.1038/s41580-020-0259-3 32636524 PMC7339799

[57] Corrò C Novellasdemunt L Vsw W. A brief history of organoids. Am J Physiol Cell Physiol (2020) 319(1):151–65. doi: 10.1152/ajpcell.00120.2020 PMC7468890 32459504

[58] Jensen C Teng Y. Is it time to start transitioning from 2D to 3D cell culture? Front Mol Biosci (2020) 7:33. doi: 10.3389/fmolb.2020.00033 32211418 PMC7067892

[59] Zhao C. Cell culture: *In vitro* model system and a promising path to *in vivo* applications. J Histotechnol (2023) 46(1):1–4. doi: 10.1080/01478885.2023.2170772 36691848

[60] Kapałczyńska M Kolenda T Przybyła W Zajączkowska M Teresiak A Filas V. 2D and 3D cell cultures – a comparison of different types of cancer cell cultures. Arch Med Sci (2016) 14(4):910–195. doi: 10.5114%2Faoms.2016.63743 30002710 10.5114/aoms.2016.63743 PMC6040128

[61] Ryu NE Lee SH Park H. Spheroid culture system methods and applications for mesenchymal stem cells. Cells NLM (Medline) (2019) 8(12):1620. doi: 10.3390/cells8121620 PMC6953111 31842346

[62] Khetan S Burdick JA. Patterning network structure to spatially control cellular remodeling and stem cell fate within 3-dimensional hydrogels. Biomaterials (2010) 31(32):8228–34. doi: 10.1016/j.biomaterials.2010.07.035 20674004

[63] Baker BM Chen CS. Deconstructing the third dimension – how 3D culture microenvironments alter cellular cues. J Cell Sci (2012) 125(13):3015–24. doi: 10.1242/JCS.079509 PMC3434846 22797912

[64] Chen SF Chang YC Nieh S Liu CL Yang CY Lin YS. Nonadhesive culture system as a model of rapid sphere formation with cancer stem cell properties. PloS One (2012) 7(2):e31864. doi: 10.1371/journal.pone.0031864 22359637 PMC3281010

[65] Mseka T Bamburg JR Cramer LP. ADF/Cofilin family proteins control formation of oriented actin-filament bundles in the cell body to trigger fibroblast polarization. J Cell Sci (2007) 120(24):4332–44. doi: 10.1242/jcs.017640 18042624

[66] Costa EC Moreira AF de Melo-Diogo D Gaspar VM Carvalho MP Correia IJ. 3D tumor spheroids: An overview on the tools and techniques used for their analysis. Biotechnol Adv (2016) 34(8):1427–41. doi: 10.1016/J.BIOTECHADV.2016.11.002 27845258

[67] Langhans SA. Three-dimensional in vitro cell culture models in drug discovery and drug repositioning. Front Pharmacol (2018) 9:883–95. doi: 10.3389/fphar.2018.00006 PMC5787088 29410625

[68] Yamada KM Doyle AD Lu J. Cell–3D matrix interactions: Recent advances and opportunities. Trends Cell Biol (2022) 32(10):883–955. doi: 10.1016/j.tcb.2022.03.002 35410820 PMC9464680

[69] Ravi M Paramesh V Kaviya SR Anuradha E Solomon FDP. 3D cell culture systems: Advantages and applications. J Cell Physiol (2015) 230(1):16–26. doi: 10.1002/JCP.24683 24912145

[70] Haisler WL Timm DM Gage JA Tseng H Killian TC Souza GR. Three-dimensional cell culturing by magnetic levitation. Nat Protoc 2013 (2013) 8(10):1940–1949. doi: 10.1038/nprot.2013.125 24030442

[71] Imamura Y Mukohara T Shimono Y Funakoshi Y Chayahara N Toyoda M. Comparison of 2D- and 3D-culture models as drug-testing platforms in breast cancer. Oncol Rep (2015) 33(4):1837–43. doi: 10.3892/or.2015.3767 25634491

[72] Hoarau-Véchot J Rafii A Touboul C Pasquier J. Halfway between 2D and animal models: Are 3D cultures the ideal tool to study cancer-microenvironment interactions? Int J Mol Sci (2018) 19(1):181. doi: 10.3390/ijms19010181 29346265 PMC5796130

[73] Barbosa MAG Xavier CPR Pereira RF Petrikaitė V Vasconcelos MH. 3D cell culture models as recapitulators of the tumor microenvironment for the screening of anti-cancer drugs. Cancers (2022) 14(1):190. doi: 10.3390/cancers14010190 PMC8749977 35008353

[74] Hickman JA Graeser R de Hoogt R Vidic S Brito C Gutekunst M. Three-dimensional models of cancer for pharmacology and cancer cell biology: Capturing tumor complexity in Vitro/Ex vivo. Biotechnol J (2014) 9(9):1115–28. doi: 10.1002/BIOT.201300492 25174503

[75] Booij TH Price LS Danen EHJ. 3D cell-based assays for drug screens: Challenges in imaging, image analysis, and high-content analysis. SLAS Discovery (2019) 24(6):615–27. doi: 10.1177/2472555219830087 30817892 PMC6589915

[76] Gao J Jung M Williams RT Hui D Russell AJ Naim AJ. Suppression of the ABCA1 cholesterol transporter impairs the growth and migration of epithelial ovarian cancer. Cancers (2022) 14(8):1878. doi: 10.3390/cancers14081878 35454786 PMC9029800

[77] Bytautaite M Petrikaite V. Comparative study of lipophilic statin activity in 2d and 3d in vitro models of human breast cancer cell lines mda-Mb-231 and mcf-7. OncoTargets Ther (2020) 13:13201–9. doi: 10.2147/OTT.S283033 PMC7769197 33380809

[78] Deezagi A Safari N. Rosuvastatin inhibit spheroid formation and epithelial–mesenchymal transition (EMT) in prostate cancer PC-3 cell line. Mol Biol Rep (2020) 47(11):8727–37. doi: 10.1007/s11033-020-05918-1 33085048

[79] Wang B Rong X Palladino END Wang J Fogelman AM Martin MG. Phospholipid remodeling and cholesterol availability regulate intestinal stemness and tumorigenesis. Cell Stem Cell (2017) 22(2):206–20. doi: 10.1016/j.stem.2017.12.017 PMC5807072 29395055

[80] Wen YA Xiong X Zaytseva YY Napier DL Vallee E Li AT. Downregulation of SREBP inhibits tumor growth and initiation by altering cellular metabolism in colon cancer article. Cell Death Dis (2018) 9(3):265. doi: 10.1038/s41419-018-0330-6 29449559 PMC5833501

[81] Kakimoto M Yamamoto H Tanaka AR. Spermine synthesis inhibitor blocks 25-Hydroxycholesterol-Induced- apoptosis *via* SREBP2 upregulation in DLD-1 cell spheroids. Biochem Biophys Rep (2020) 22:100754. doi: 10.1016/j.bbrep.2020.100754 32258442 PMC7109571

[82] Aguirre-Portolés C Feliu J Reglero G de Molina AR. ABCA1 overexpression worsens colorectal cancer prognosis by facilitating tumour growth and caveolin-1-Dependent invasiveness, and these effects can be ameliorated using the BET inhibitor apabetalone. Mol Oncol (2018) 12(10):1735–52. doi: 10.1002/1878-0261.12367 PMC6166002 30098223

[83] Guerra FS Sampaio LS Konig S Bonamino M Rossi MID Costa ML. Membrane cholesterol depletion reduces breast tumor cell migration by a mechanism that involves non-canonical wnt signaling and IL-10 secretion. Trans Med Commun (2016) 1(1):3. doi: 10.1186/s41231-016-0002-4

[84] Namba Y Sogawa C Okusha Y Kawai H Itagaki M Ono K. Depletion of lipid efflux pump ABCG1 triggers the intracellular accumulation of extracellular vesicles and reduces aggregation and tumorigenesis of metastatic cancer cells. Front Oncol (2018) 8:376. doi: 10.3389/fonc.2018.00376 30364132 PMC6191470

[85] Guillaumond F Bidaut G Ouaissi M Servais S Gouirand V Olivares O. Cholesterol uptake disruption, in association with chemotherapy, is a promising combined metabolic therapy for pancreatic adenocarcinoma. Proc Natl Acad Sci USA (2015) 112(8):2473–8. doi: 10.1073/pnas.1421601112 PMC4345573 25675507

[86] Gong J Sachdev E Robbins LA Lin E Hendifar AE Mita MM. Statins and pancreatic cancer. Oncol Lett (2017) 13(3):1035–40. doi: 10.3892/ol.2017.5572 PMC5403279 28454210

[87] Chen WCY Boursi B Mamtani R Yang YX. Total serum cholesterol and pancreatic cancer: A nested case-control study. Cancer Epidemiol Biomarkers Prev (2019) 28(2):363–9. doi: 10.1158/1055-9965.EPI-18-0421 30333217

[88] Shinkawa T Ohuchida K Mochida Y Sakihama K Iwamoto C Abe T. Subtypes in pancreatic ductal adenocarcinoma based on niche factor dependency show distinct drug treatment responses. J Exp Clin Cancer Res (2022) 41(1):89. doi: 10.1186/s13046-022-02301-9 35272688 PMC8908673

[89] Oni TE Biffi G Baker LA Hao Y Tonelli C Somerville TDD. SOAT1 promotes mevalonate pathway dependency in pancreatic cancer. J Exp Med (2020) 217(9):e20192389. doi: 10.1084/jem.20192389 32633781 PMC7478739

[90] Nicolle R Blum Y Marisa1 L Loncle C Gayet O Moutardier V. Pancreatic adenocarcinoma therapeutic targets revealed by tumor-stroma cross-talk analyses in patient-derived xenografts rémy. Cell Rep (2017) 21(9):2458–70. doi: 10.1016/j.celrep.2017.11.003 PMC6082139 29186684

[91] Yang J Broman MM Cooper PO Lanman NA Douglas W Morrissey C. Distinct expression patterns of SULT2B1b in human prostate epithelium. Prostate (2019) 79(11):1256–66. doi: 10.1002/pros.23829 PMC7064034 31212370

[92] Wang Z Gerstein M Snyder M. RNA-Seq: A revolutionary tool for transcriptomics zhong. Nat Rev Genet (2009) 10(1):57–63. doi: 10.1038/nrg2484 PMC2949280 19015660

[93] Muñoz-Galindo L Melendez-Zajgla J Pacheco-Fernández T Rodriguez-Sosa M Mandujano-Tinoco EA Vazquez-Santillan K. Changes in the transcriptome profile of breast cancer cells grown as spheroids. Biochem Biophys Res Commun (2019) 516(4):1258–64. doi: 10.1016/j.bbrc.2019.06.155 31301772

[94] Ehmsen S Pedersen MH Wang G Terp MG Arslanagic A Hood BL. Increased cholesterol biosynthesis is a key characteristic of breast cancer stem cells influencing patient outcome. Cell Rep (2019) 27(13):3927–38. doi: 10.1016/j.celrep.2019.05.104 31242424

[95] Dattilo R Mottini C Camera E Lamolinara A Auslander N Doglioni G. Pyrvinium pamoate induces death of triple-negative breast cancer stem–like cells and reduces metastases through effects on lipid anabolism. Cancer Res (2022) 80(19):4087–102. doi: 10.1158/0008-5472.CAN-19-1184 PMC8808379 32718996

[96] Seo Y Kim J Park SJ Park JJ Cheon JH Kim WH. Metformin suppresses cancer stem cells through ampk activation and inhibition of protein prenylation of the mevalonate pathway in colorectal cancer. Cancers (2020) 12(9):2554. doi: 10.3390/cancers12092554 32911743 PMC7563617

[97] He L Li H Pan C Hua Y Peng J Zhou Z. Squalene epoxidase promotes colorectal cancer cell proliferation through accumulating calcitriol and activating CYP24A1-mediated MAPK signaling. Cancer Commun (2021) 41(8):726–46. doi: 10.1002/cac2.12187 PMC8360641 34268906

[98] Jun SY Brown AJ Chua NK Yoon JY Lee JJ Yang JOK. Reduction of squalene epoxidase by cholesterol accumulation accelerates colorectal cancer progression and metastasis. Gastroenterology (2021) 160(4):1194–207. doi: 10.1053/j.gastro.2020.09.009 32946903

[99] Huang B Song Bl Xu C. Cholesterol metabolism in cancer: Mechanisms and therapeutic opportunities. Nat Metab (2020) 2(2):132–41. doi: 10.1038/s42255-020-0174-0 32694690

[100] Gao S Soares F Wang S Wong CC Chen H Yang Z. CRISPR screens identify cholesterol biosynthesis as a therapeutic target on stemness and drug resistance of colon cancer. Oncogene (2021) 40(48):6601–13. doi: 10.1038/s41388-021-01882-7 PMC8639446 34621019

[101] Rodrigues D Herpers B Ferreira S Jo H Fisher C Coyle L. A transcriptomic approach to elucidate the mechanisms of gefitinib-induced toxicity in healthy human intestinal organoids. Int J Mol Sci (2022) 23(4):2213. doi: 10.3390/ijms23042213 35216325 PMC8876167

[102] Neuwirt H Bouchal J Kharaishvili G Ploner C Jöhrer K Pitterl F. Cancer-associated fibroblasts promote prostate tumor growth and progression through upregulation of cholesterol and steroid biosynthesis. Cell Commun Signaling (2020) 18(1):11. doi: 10.1186/s12964-019-0505-5 PMC6979368 31980029

[103] Jin U Park SJ Park SM. Cholesterol metabolism in the brain and its association with parkinson’s disease. Exp Neurobiol (2019) 28(5):554–67. doi: 10.5607/en.2019.28.5.554 31698548 PMC6844833

[104] Shakya S Gromovsky AD Hale JS Knudsen AM Prager B Wallace LC. Altered lipid metabolism marks glioblastoma stem and non-stem cells in separate tumor niches. Acta Neuropathol Commun (2021) 9(1):101. doi: 10.1186/s40478-021-01205-7 34059134 PMC8166002

[105] Lewis CA Brault C Peck B Bensaad K Griffiths B Mitter R. SREBP maintains lipid biosynthesis and viability of cancer cells under lipid- and oxygen-deprived conditions and defines a gene signature associated with poor survival in glioblastoma multiforme. Oncogene (2015) 34(40):5128–40. doi: 10.1038/onc.2014.439 25619842

[106] Kim HY Kim DK Bae SH Gwak HR Jeon JH Kim JK. Farnesyl diphosphate synthase is important for the maintenance of glioblastoma stemness. Exp Mol Med (2018) 50(10):1–12. doi: 10.1038/s12276-018-0166-2 PMC6193020 30333528

[107] Liu M Xia Y Ding J Ye B Zhao E Choi J-H. Transcriptional profiling reveals a common metabolic program for tumorigenicity in high-risk human neuroblastoma and mouse neuroblastoma sphere-forming cells. Cell Reports (2016) 17(2):609–23. doi: 10.1016/j.celrep.2016.09.021 PMC5536348 27705805

[108] AhmadiMAmiriSPecicSMachajFRosikJMarekJ. Pleiotropic effects of statins: A focus on cancer. Biochim Biophys Acta - Mol Basis Dis (2020) 1866(12):165968. doi: 10.1016/j.bbadis.2020.165968 32927022

[109] Zhang ZY Zheng SH Yang WG Yang C Yuan WT. Targeting colon cancer stem cells with novel blood cholesterol drug pitavastatin. Eur Rev Med Pharmacol Sci (2017) 21(6):1226–33. 28387909

[110] Norkin M Ordóñez-Morán P Huelsken J. High-content, targeted RNA-seq screening in organoids for drug discovery in colorectal cancer. Cell Rep (2021) 35(3):109026. doi: 10.1016/j.celrep.2021.109026 33882314

[111] Alzeeb G Arzur D Trichet V Talagas M Corcos L Jossic-Corcos CL. Gastric cancer cell death analyzed by live cell imaging of spheroids. Sci Rep (2022) 12(1):1488. doi: 10.1038/s41598-022-05426-1 35087119 PMC8795446

[112] Cai D Wang J Gao B Li J Wu F Zou JX. RORγ is a targetable master regulator of cholesterol biosynthesis in a cancer subtype. Nat Commun (2019) 10(1):4621. doi: 10.1038/s41467-019-12529-3 31604910 PMC6789042

[113] Varan G Akkın S Demirtürk N Benito JM Bilensoy E. Erlotinib entrapped in cholesterol-depleting cyclodextrin nanoparticles shows improved antitumoral efficacy in 3D spheroid tumors of the lung and the liver. J Drug Targeting (2021) 29(4):439–53. doi: 10.1080/1061186X.2020.1853743 33210947

[114] Acier A Godard M Gassiot F Finetti P Rubis M Nowak J. LDL receptor-peptide conjugate as *in vivo* tool for specific targeting of pancreatic ductal adenocarcinoma. Commun Biol (2021) 4(1):987. doi: 10.1038/s42003-021-02508-0 34413441 PMC8377056

[115] Lee B Park SJ Lee S Lee J Lee E Yoo ES. Lomitapide, a cholesterol-lowering drug, is an anticancer agent that induces autophagic cell death *via* inhibiting MTOR. Cell Death Dis (2022) 13(7):603. doi: 10.1038/s41419-022-05039-6 35831271 PMC9279289

[116] Coisne C Tilloy S Monflier E Wils D Fenart L Gosselet. F. Cyclodextrins as emerging therapeutic tools in the treatment of cholesterol-associated vascular and neurodegenerative diseases. Molecules (2016) 21(12):1748. doi: 10.3390/molecules21121748 27999408 PMC6273856

[117] Mohammad N Malvi P Meena AS Singh SV Chaube B Vannuruswamy G. Cholesterol depletion by methyl-β-Cyclodextrin augments tamoxifen induced cell death by enhancing its uptake in melanoma. Mol Cancer (2014) 13(1):204. doi: 10.1186/1476-4598-13-204 25178635 PMC4175626

[118] Yang J McDowell A Kim EK Seo H Lee WH Moon CM. Development of a colorectal cancer diagnostic model and dietary risk assessment through gut microbiome analysis. Exp Mol Med (2019) 51(10):1–15. doi: 10.1038/s12276-019-0313-4 PMC6802675 31582724

[119] Salinas ML Fuentes NR Choate R Wright RC McMurray DN Chapkin RS. AdipoRon attenuates wnt signaling by reducing cholesterol-dependent plasma membrane rigidity. Biophys J (2020) 118(4):885–97. doi: 10.1016/j.bpj.2019.09.009 31630812 PMC7036725

[120] Chen N Wang J. Wnt/β-catenin signaling and obesity. Front Physiol (2018) 9:792. doi: 10.3389/fphys.2018.00792 30065654 PMC6056730

[121] Carrer A Trefely S Zhao S Campbell SL Norgard RJ Schulz KC. Acetyl-CoA metabolism supports multi-step pancreatic tumorigenesis. Physiol Behav (2017) 9(3):416–35. doi: 10.1158/2159-8290.CD-18-0567 PMC6643997 30626590

[122] Morey P Pfannkuch L Pang E Boccellato F Sigal M Imai-Matsushima A. Helicobacter pylori depletes cholesterol in gastric glands to prevent interferon gamma signaling and escape the inflammatory response. Gastroenterology (2018) 154(5):1391–404.e9. doi: 10.1053/j.gastro.2017.12.008 29273450

[123] Yoshida T Kato J Inoue I Yoshimura N Deguchi H Mukoubayashi C. Cancer development based on chronic active gastritis and resulting gastric atrophy as assessed by serum levels of pepsinogen and helicobacter pylori antibody titer. Int J Cancer (2014) 134(6):1445–57. doi: 10.1002/ijc.28470 24009139

[124] Li C Wang Y Liu D Wong CC Coker OO Zhang X. Squalene epoxidase drives cancer cell proliferation and promotes gut dysbiosis to accelerate colorectal carcinogenesis. Gut (2022) 71:2253–65. doi: 10.1136/gutjnl-2021-325851 PMC9554078 35232776

[125] Sato T Clevers H. Growing self-organizing mini-guts from a single intestinal stem cell: Mechanism and applications. Science (2013) 340(6137):1190–4. doi: 10.1126/science.1234852 23744940

[126] Demchenko A Lavrov A Smirnikhina S. Lung organoids: Current strategies for generation and transplantation. Cell Tissue Res (2022) 390(3):317–33. doi: 10.1007/s00441-022-03686-x PMC9522545 36178558

[127] Sun XY Ju XC Li Y Zeng PM Wu J Zhou YY. Generation of vascularized brain organoids to study neurovascular interactions. ELife (2022) 11. doi: 10.7554/eLife.76707 PMC9246368 35506651

[128] Lee J van der Valk WH Serdy SA Deakin CC Kim J Le AP. Generation and characterization of hair-bearing skin organoids from human pluripotent stem cells. Nat Protoc 2022 (2022) 17(5):1266–305. doi: 10.1038/s41596-022-00681-y PMC10461778 35322210

[129] Ludwig AL Mayerl SJ Gao Y Banghart M Bacig C Fernandez Zepeda MA. Re-formation of synaptic connectivity in dissociated human stem cell-derived retinal organoid cultures. Proc Natl Acad Sci United States America (2023) 120(2):e2213418120. doi: 10.1073/pnas.2213418120 PMC9926218 36598946

[130] Kim S Min S Choi YS Jo SH Jung JH Han K. Tissue extracellular matrix hydrogels as alternatives to matrigel for culturing gastrointestinal organoids. Nat Commun (2022) 13(1):1692. doi: 10.1038/s41467-022-29279-4 35354790 PMC8967832

[131] Suarez-Martinez E Suazo-Sanchez I Celis-Romero M Carnero A. 3D and organoid culture in research: Physiology, hereditary genetic diseases and cancer. Cell Biosci (2022) 12(1):39. doi: 10.1186/s13578-022-00775-w 35365227 PMC8973959

[132] Torres-Quesada O Doerrier C Strich S Gnaiger E Stefan E. Physiological cell culture media tune mitochondrial bioenergetics and drug sensitivity in cancer cell models. Cancers (2022) 14(16):3917. doi: 10.3390/cancers14163917 36010911 PMC9405899

